# Analysis of Site Formation and Assemblage Integrity Does Not Support Attribution of the Uluzzian to Modern Humans at Grotta del Cavallo

**DOI:** 10.1371/journal.pone.0131181

**Published:** 2015-07-08

**Authors:** João Zilhão, William E. Banks, Francesco d’Errico, Patrizia Gioia

**Affiliations:** 1 Universitat de Barcelona, Seminari d'Estudis i Recerques Prehistòriques (SGR2014-00108), Departament de Prehistòria, Història Antiga i Arqueologia, Facultat de Geografia i Història, C/ Montalegre 6, 08001 Barcelona, Spain; 2 Institució Catalana de Recerca i Estudis Avançats (ICREA), Passeig Lluís Companys 23, 08010 Barcelona, Spain; 3 Unité Mixte de Recherche 5199, de la Préhistoire à l’Actuel: Culture, Environnement et Anthropologie (UMR 5199 – PACEA), Centre National de la Recherche Scientifique (CNRS), Université de Bordeaux, Bâtiment B18, Allée Geoffroy Saint Hilaire, CS 50023 33615 Pessac Cedex, France; 4 Biodiversity Institute, University of Kansas, 1345 Jayhawk Blvd., Dyche Hall, Lawrence, KS, 66045–7562, United States of America; 5 Department of Archaeology, History, Cultural Studies and Religion, University of Bergen, Øysteinsgate 3, 5007 Bergen, Norway; 6 Sapienza—Università di Roma, Dipartimento di Scienze dell’Antichità, Facoltà di Lettere e Filosofia, Piazzale Aldo Moro 5, 00185 Rome, Italy; New York State Museum, UNITED STATES

## Abstract

Based on the morphology of two deciduous molars and radiocarbon ages from layers D and E of the Grotta del Cavallo (Lecce, Italy), assigned to the Uluzzian, it has been proposed that modern humans were the makers of this Early Upper Paleolithic culture and that this finding considerably weakens the case for an independent emergence of symbolism among western European Neandertals. Reappraisal of the new dating evidence, of the finds curated in the Taranto Antiquities depot, and of coeval publications detailing the site’s 1963–66 excavations shows that (a) Protoaurignacian, Aurignacian and Early Epigravettian lithics exist in the assemblages from layers D and E, (b) even though it contains both inherited and intrusive items, the formation of layer D began during Protoaurignacian times, and (c) the composition of the extant Cavallo assemblages is influenced in a non-negligible manner by the post-hoc assignment of items to stratigraphic units distinct from that of original discovery. In addition, a major disturbance feature affected the 1960s excavation trench down to Mousterian layer F, this feature went unrecognized until 1964, the human remains assigned to the Uluzzian were discovered that year and/or the previous year, and there are contradictions between field reports and the primary anthropological description of the remains as to their morphology and level of provenience. Given these major contextual uncertainties, the Cavallo teeth cannot be used to establish the authorship of the Uluzzian. Since this technocomplex’s start date is ca. 45,000 calendar years ago, a number of Neandertal fossils are dated to this period, and the oldest diagnostic European modern human fossil is the <41,400 year-old Oase 1 mandible, Neandertal authorship of the Uluzzian remains the parsimonious reading of the evidence.

## Introduction

### Background, outline and aims

In Europe, the Middle-to-Upper Paleolithic transition took place in an interval of about ten millennia that begins ca. 47,500 years ago with continent-wide Neandertal populations manufacturing Mousterian stone tool assemblages and ends ca. 37,500 years ago with continent-wide anatomically modern populations manufacturing Aurignacian stone tool assemblages. A number of regionally diverse, stratigraphically intermediate technocomplexes, so-called “transitional,” occupy the intervening millennia. Even though they sometimes feature traits inherited from the Middle Paleolithic, these transitional entities are defined by the presence of at least some key components of the so-called “Upper Paleolithic package” (e.g., blade and bladelet production, objects of personal ornamentation, bone tools). As many of these innovations are widely acknowledged to represent reliable material cultural proxies for symbolic thinking, the authorship—Neandertal, or modern human—of the transitional technocomplexes in which they are observed for the first time in Europe has been the matter of much controversy ([1] and references therein).

**Fig 1:**
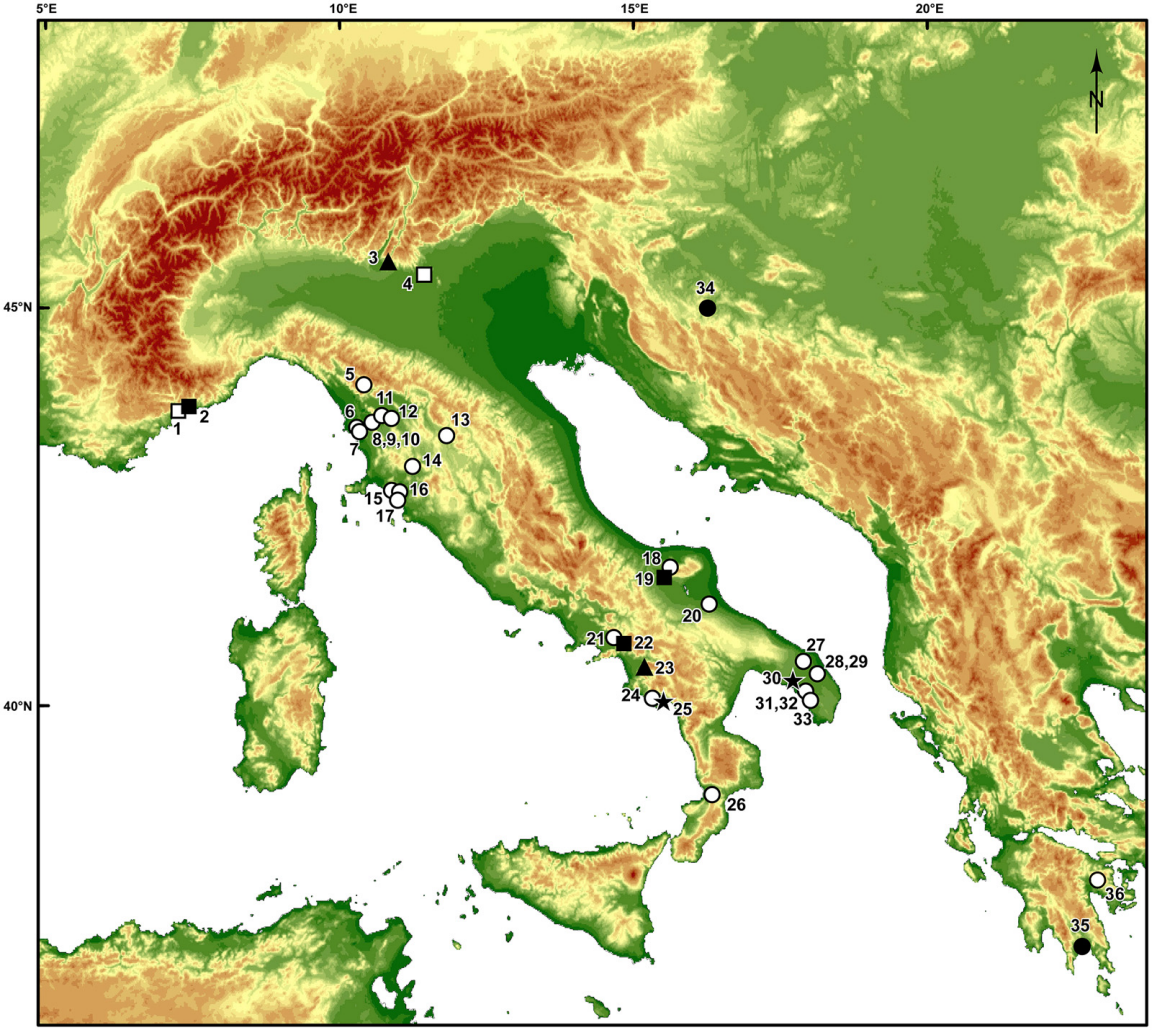
Site locations. Open circles: Uluzzian sites; Solid circles: Neandertal remains dated to the Uluzzian time range. Open squares: Protoaurignacian sites; Solid squares: Protoaurignacian sites used in the age model of ref. [14]. Solid triangles: Sites with Uluzzian and Protoaurignacian levels used in the age model of ref. [14]. Stars: Grotta del Cavallo and Grotta del Poggio, sites from which come the teeth published in ref. [15]. List of indicated sites: 1. Grotte de l’Observatoire; 2. Grimaldi sites (e.g. Mochi); 3. Fumane; 4. Paina; 5. Porcari; 6. Salviano; 7. Maroccone; 8. Val di Cava; 9. Podere Colline; 10. Casa ai Pini; 11. San Romano; 12. San Leonardo; 13. Indicatore; 14. Santa Lucia I; 15. Val Berretta; 16. Calvello; 17. Fabbrica; 18. Foresta Umbra; 19. Paglicci; 20. Falce del Viaggio; 21. Tornola; 22. Serino; 23. Castelcivita; 24. Cala; 25. Poggio; 26. San Pietro; 27. Torre Testa; 28. Cosma; 29. Uluzzo; 30. Cavallo; 31. Mario Bernardini; 32. Serra Cicora; 33. Veneri de Parabita; 34. Vindija; 35. Lakonis I; 36. Klisoura 1. Base map created from ETOPO1 Global Relief Model data (http://www.ngdc.noaa.gov/mgg/global/).

At the heart of the problem lies these transitional contexts’ scarcity of human remains; indeed, most (e.g., the Bohunician of Moravia) have yielded none. Neandertal affinity has been suggested for two deciduous molars attributed to layer E of Grotta del Cavallo [2], whose stone tool assemblages belong to the Uluzzian, a transitional technocomplex found in Italy and Greece [3–6] (Fig 1), and the stratigraphic association of diagnostic fossils with diagnostic lithics at the sites of Saint-Césaire and Grotte du Renne supports Neandertal authorship of the French Châtelperronian [7–9]. Recently, however, the validity of the Neandertal-Châtelperronian association has been questioned [10,11], and reanalysis of the Cavallo teeth with new techniques suggested that they were modern human rather than Neandertal [12]. Based on these reassessments of the evidence and on new radiocarbon results placing the emergence of the Uluzzian ca. 45,000 years ago, the authors of the new Cavallo study concluded that this technological tradition was produced by modern humans arriving in Southern Europe three to four millennia earlier than previously thought [12,13]; they further suggested that, consequently, it would now be “much less likely that Neanderthals developed their own Upper Palaeolithic suite of behaviours before the arrival of anatomically modern humans” [12] (p. 528).

Such views have not gone unchallenged [16], but their broader paleoanthropological significance and recent reassertions [17,18] make it necessary that the Cavallo evidence be carefully scrutinized with an eye towards clarifying the key issue, that being the reliability of the association between the fossils and the Uluzzian. This issue has two facets: one concerns the definition and homogeneity of the artefact assemblages retrieved alongside the teeth; the other concerns the integrity of the deposit, namely whether we can rule out the possibility that the teeth represent material derived from under- or overlying archaeological horizons. All of this has implications not only for the age of the human remains but also for the chronology of the technocomplex, as it has a bearing on whether the dated samples relate to the Uluzzian and reflect its age at the site.

As described in the Materials and Methods section, we address these issues via the analysis of a body of data comprised of: (a) the contents of coeval excavation reports; (b) a mid-1980s record of the technology, typology, dimensions, provenience and labeling of the totality of the Cavallo lithics that, at the time, had been assigned to the Uluzzian; (c) our hands-on analysis of the collection in storage at the Taranto depot of Apulia’s Direction of Antiquities and of the associated archives.

### Grotta del Cavallo: location, excavation history and assemblages

**Fig 2:**
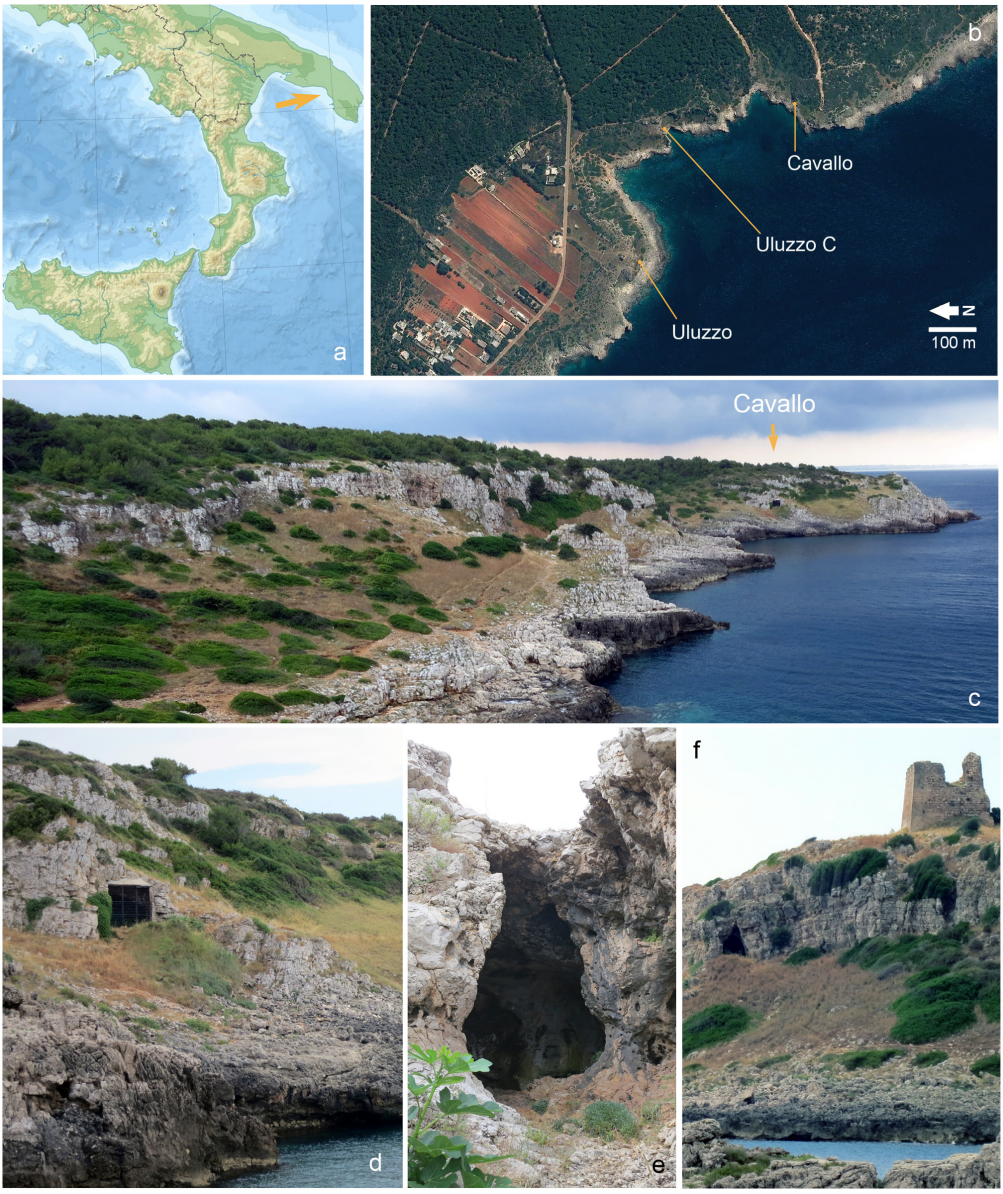
Grotta del Cavallo and the Uluzzo Bay sites. **a.** location of Uluzzo Bay on a physical map of southern Italy; **b.** Satellite view of the Uluzzo Bay (http://www.pcn.minambiente.it/GN/), with indication of the Uluzzian sites discussed in the text; **c.** the setting of the Grotta del Cavallo (photographed from the West); **d.** the Grotta del Cavallo entrance; **e.** the Grotta di Uluzzo entrance; **f.** the setting of the Grotta di Uluzzo (photographed from the Northwest).

The Grotta del Cavallo (Santa Caterina, Nardò, Lecce; 40°9’19.00” N; 17°57’37.90” E; Fig 2) was first excavated in the early 1960s by Arturo Palma di Cesnola, a leading figure of the Paleolithic Archeology of Italy. Born in Florence in 1928, Palma di Cesnola was appointed Professor of Human Paleontology at the University of Siena in 1968, a post he held until retirement, 30 years later. Early on in his career, he developed an interest in the cave sites of southern Italy that, eventually, led him to the Bay of Uluzzo. Supported by the Florence-based *Istituto Italiano di Preistoria e Protostoria* and the Provincial Archeological Museum of Lecce, Palma di Cesnola’s work in this area was prompted by a speleologist’s discovery, in 1960, of Middle and Upper Paleolithic stone tools at a number of sites situated around the bay and the adjacent hinterland.

Palma di Cesnola took up responsibility for the excavations at Cavallo, and at the same time his University of Florence colleague Edoardo Borzatti von Löwenstern directed those carried out at the nearby sites of Grotta di Uluzzo (40°9’30.27” N; 17°57’24.18” E) and Grotta-Riparo Uluzzo C (40°9’28.31” N; 17°57’35.56” E) (Fig 2). Where Cavallo is concerned, published accounts [19–30] state that: (a) after initial testing, in 1961, excavations were carried out annually between 1963 and 1966 and interrupted in 1967; (b) in the years that followed, the site was extensively damaged by the activity of looters; and (c) in the late 1970s and early 1980s, after several years spent repairing the destruction, archeological investigations resumed and remnants of the Uluzzian deposit spared by the looting were identified and excavated. Subsequent and ongoing work [31–33] focused on the Mousterian sequence below the Uluzzian, which had been probed in the 1960s down to the basal Tyrrhenian beach, ca. 7 m below surface.

**Fig 3:**
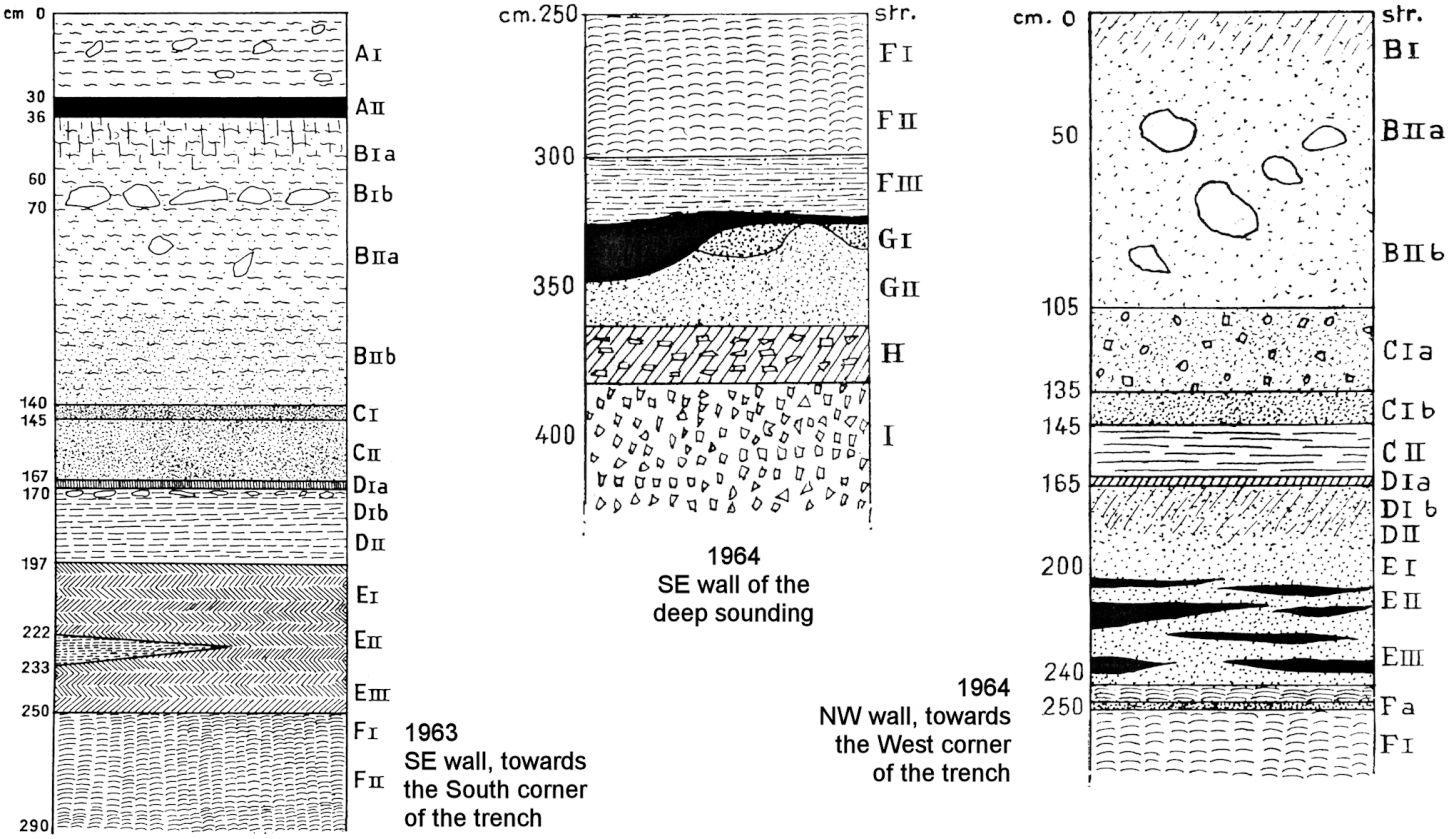
The Grotta del Cavallo stratigraphy according to Palma di Cesnola. The upper part of the Cavallo deposit as understood in the field seasons (1963–64) during which the human teeth were discovered. The different stratigraphic units recognized were designated by the excavator as follows: layer A, Neolithic; layer B, Romanellian (= Late Epigravettian); layer C, volcanic ash lens; layers D–E, Uluzzian; layers F–I, Mousterian. Reproduced from Fig 2 of ref. [19] and Figs 1–2 of ref. [20].

The Uluzzian deposit of Cavallo is sandwiched between Mousterian layer F and the ca. 1 m-thick layer B; the latter has been assigned to the Romanellian (= Late Epigravettian) and is overlain by a surficial and disturbed Holocene layer A. Palma di Cesnola described the Uluzzian sequence as: (a) a lower layer of silty sands extensively blackened due to its richness in combustion byproducts—layer E, ca. 50 cm-thick, subdivided into level E-III at the base and level E-II/I at the top; (b) an upper, brown-reddish layer of similar texture—layer D, ca. 30 cm-thick, subdivided into level D-II at the base and level D-I at the top (Fig 3); (c) an intermediate level E/D, created in 1964 to accommodate the indistinctiveness of the boundary between layers E and D then observed in parts of the trench. Note that the “level” subdivisions were not stratigraphic units recognized as such at the time of excavation. Rather, they correspond to the grouping together for analytical purposes of a number of horizontal spits of arbitrary thickness; these ca. 10 cm-thick *tagli* (designated D1-D4 and E1-E7) were the units of excavation actually used in the field [23].

Palma di Cesnola published preliminary assessments of the stone tool assemblages from Cavallo in reports concerning the first two years of excavation, 1963 and 1964 [19,20], followed in 1965 by his own monographic study of the Uluzzian levels [21,23]. At the time, this material was also studied by G. Laplace [34]. Some twenty years later, the collection was reanalyzed in a Bordesian framework by one of us (PG) as part of a Ph.D. dissertation on the Uluzzian of Italy [35–37]. The total number of Laplacian “primary types” counted by Palma di Cesnola is 1181 [21,23], and the total number of “retouched tools” counted by PG is 983 [35,36]. Given that multiple tools are counted as single items in a Bordesian framework but as two (or more) types in the Laplace system, the broad equivalence of these counts suggests that the collection made available to PG for study was the same that Palma di Cesnola analyzed in 1965, and, therefore, that it included neither the finds recovered in 1966 nor those from the 1970s and the 1980s. Alternatively, the equivalence may indicate that such later finds were, by comparison, of limited abundance, but no certainty is possible.

Here, our technological and typological discussion of the Cavallo assemblages is restricted to the material that one can securely assign to the first two years of excavation, based on coeval publication by the excavator himself [19–24] and the records kept in the Archaeological Museum and the Antiquities depot of Taranto. The reason being that such material constitutes the immediate archeological context of the human remains assigned to the Uluzzian, first published in 1967 [15] but recovered in 1963–64 (see below).

## Results and Discussion

### The Aurignacian lithics

**Fig 4:**
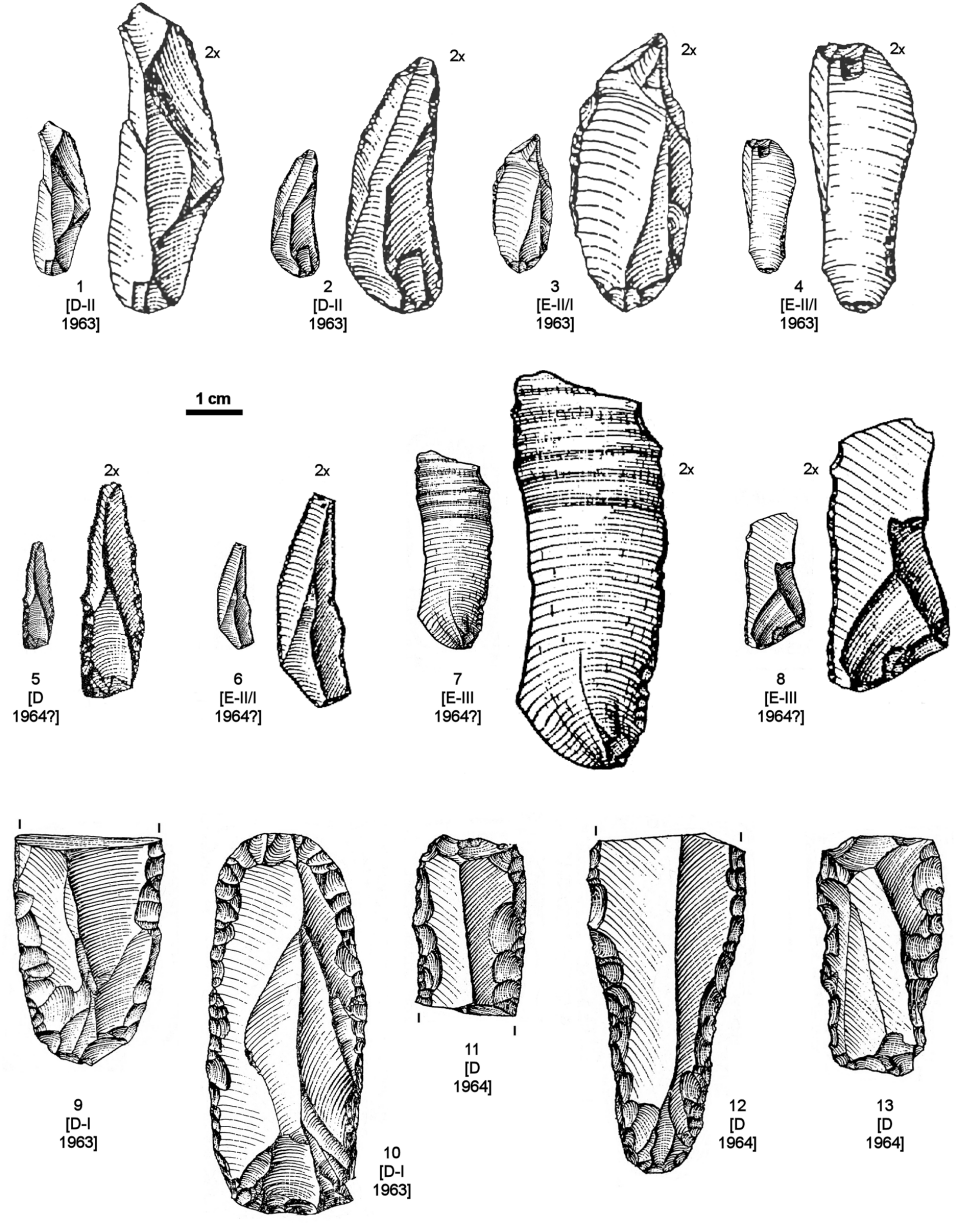
Aurignacian/Protoaurignacian diagnostics from Grotta del Cavallo. Diagnostic Aurignacian or Protoaurignacian stone tools recovered from layers D and E in the 1963–64 field seasons, those during which the human teeth were discovered. For each item, the level of provenience and year of discovery are indicated. 1–8: Dufour bladelets; 9, 11–13: retouched blades; 10: endscraper on a retouched blade. Reproduced from refs. [19,21,23].

From the beginning, Palma di Cesnola noted that significant variation existed in the composition of the lithic assemblages as one moved up in the sequence of stratigraphic units that he assigned to the Uluzzian. His interpretation was that the differences resulted from change through time, leading to the emergence in layer D of typological and technological features elsewhere characteristic of the Aurignacian [3,21,23]. The objects bearing such Aurignacian features in a more readily apparent manner were subsequently interpreted by one of us (PG) as reflecting the presence of a truly Aurignacian component in the assemblage from layer D [36–38], which would therefore be mixed instead of solely Uluzzian [16,39]. These views have since been challenged on typological grounds [18]. The key issue, however, is of a technological nature, as demonstrated by the items in Fig 4.

The four artefacts in the upper row of Fig 4 are reproduced from the 1963 report [19], while those in the middle row appear for the first time in the 1965–66 monographic study of the material [21,23]. Even though largely ignored in the literature dealing with the Uluzzian levels of the site (or the Italian Uluzzian in general), these items are typologically unambiguous, and the excavator clearly saw them for what they are: Dufour bladelets, as indicated by their size, blank type (the dorsal scar pattern in nos. 1–3 and 5–6 suggests detachment from carinated or nosed scrapers/cores, as does the curvature and apparent twisting of nos. 1–2 and 6), and marginal retouch—inverse in two cases, but direct (as is often the case in the Protoaurignacian and the Aurignacian of Italy [4,40]) in the other six. “Dufour bladelet” is the expression actually used by Palma di Cesnola for nos. 1–4; for nos. 5–7 he used “marginally backed bladelet,” a terminology that reflects the Laplacian framework of his 1965–66 study rather than a different classification. Of these eight Dufour bladelets, three are from layer D (in two cases, more specifically from level D-II), another three from level E-II/I, and two from level E-III.

Palma di Cesnola’s descriptions of the material are consistent with this typological classification. When discussing blade/bladelet production at the site, he began, with respect to layer E, by writing that level E-III contained “only nine bladelets, mostly of siliceous limestone, rather irregular in shape, bearing a minute peripheral retouch, more invasive in two others” [21] (p. 52). He then added that level E-II/I contained “marginally backed bladelets: eight pieces, of which six under 30 mm in length and two between 30 and 40 mm. The retouch, at times very minute, is nearly direct in four: total and bilateral in one (…), it spares the distal part of the bladelet in another, and is proximal in the remaining two (…). Retouch is inverse on three bladelets (total in one case, proximal in two) and alternate on a single specimen (distal and proximal).” [23] (p. 12).

Where these descriptions are referred to illustrations, there can be no doubt that most if not all of the items in question are indeed Dufour bladelets and, therefore, that those in our Fig 4 represent a minimum number only. To be conservative, we will use this minimum number in our discussion of the Cavallo assemblages, but the counts in Palma di Cesnola’s 1965–66 monographic study of the material corroborate that more exist indeed among the stone tools assigned to layers E and D: the total given in the summary table [23] (p. 54) for Laplacian types Pd1 and Ld1 (where Laplace himself placed Dufour bladelets in the Protoaurignacian and Aurignacian assemblages that he studied at that time, e.g. those from Gatzarria [41]) is 20—four in layer D, three in level E/D, eight in level E-II/I, and five in level E-III; and the total obtained adding-up the “marginally backed bladelets” in the level-by-level discussion of the finds is 21, due to an additional specimen from layer D.

The lower part of Fig 4 (nos. 8–12) illustrates blade tools from layer D made on large, elongated blanks with regular dorsal ridges. The dimensions of the complete endscraper on a retouched blade (no. 10) are 6.6×2.8 cm. The blade with Aurignacian retouch (no. 12) is a 5.8 cm-long fragment of similar width and whose dorsal scar pattern suggests that the break lies at approximately half the original length (which, therefore, would have been at least 11–12 cm). Palma di Cesnola fully realized the special nature of this material. In his monographic discussion of the layer D assemblage he noted that, alongside bladelet tools similar to those found in layer E, something entirely new appeared for the first time [23] (p. 27): “a group of blades, of quite larger size (up to more than 60 mm), bear a more prominent retouch, uni- and bilaterally,” including “specimens that, despite their fragmented condition, display a regularity in the detachment from the core and the processing of the edges fully unknown in either the Archaic or the Middle Uluzzian” (i.e., in either level E-III or level E-II/I). Commenting on two of the items reproduced in our Fig 4, he added: concerning no. 11, that “the retouch is particularly invasive and flat”; and, concerning no. 12, that this “remarkable specimen (…) features bilateral retouch, forming half-way through a slight narrowing and evoking the so-called ‘lames étranglées’ of the classical Aurignacian.” Finally, he recalled that, in 1963, 11 blades “with more or less typical aurignacoid traits” were collected in layer D, and provides as corroborating illustrations nos. 9–10 of our Fig 4.

The size and technological features of these blade blanks are inconsistent with the reduction system represented by the core types found in the Uluzzian, be it at Cavallo or elsewhere. Suffice is to quote here how Palma di Cesnola described Uluzzian technology in his last monographic assessment of the technocomplex as a whole [3]: “Cores are of small size and not very regular. Unidirectional types tend to predominate, but opposed platform cores also exist, especially in those contexts where bipolar reduction is widely used and the production of very small flakes is important. In such cases, some authors have even talked about scaled cores or forms of passage between cores and splintered pieces. Multidirectional polyhedric cores and, more rarely, discoid ones, are also characteristic (…). Prismatic or subprismatic blade and bladelet cores are extremely rare. (…) The laminarity of the Uluzzian industry is very low (…) Uluzzian blades are in general rather short (often not much longer than flakes) and triangular in cross-section. As with flakes, their dorsal side often features areas where cortex is still apparent.”

Subsequent technological analyses of Uluzzian lithic production [42] have corroborated these insights. They have also shown, e.g. at Fumane, that the Uluzzian changed through time in a direction opposite that initially suggested for Cavallo by Palma di Cesnola; namely, by losing the elements of Levallois laminarity inherited from the preceding Mousterian and becoming exclusively focused on the extraction of flake blanks—not by acquiring, in its later stages, elements of Aurignacian-like, prismatic core blade/bladelet production.

A technological system such as that just described for the Uluzzian could not have produced the blade blanks illustrated in Fig 4. The conclusion that they are unrelated to the Uluzzian is further corroborated by the conspicuous absence of similar material in the corresponding assemblages from other Italian sites where the technocomplex is represented, namely La Fabbrica or Castelcivita [43]. Conversely, the affinities of these large blade tools with modes of production and edge retouch that are well documented in the Aurignacian indicate that such is their true cultural filiation.

**Fig 5:**
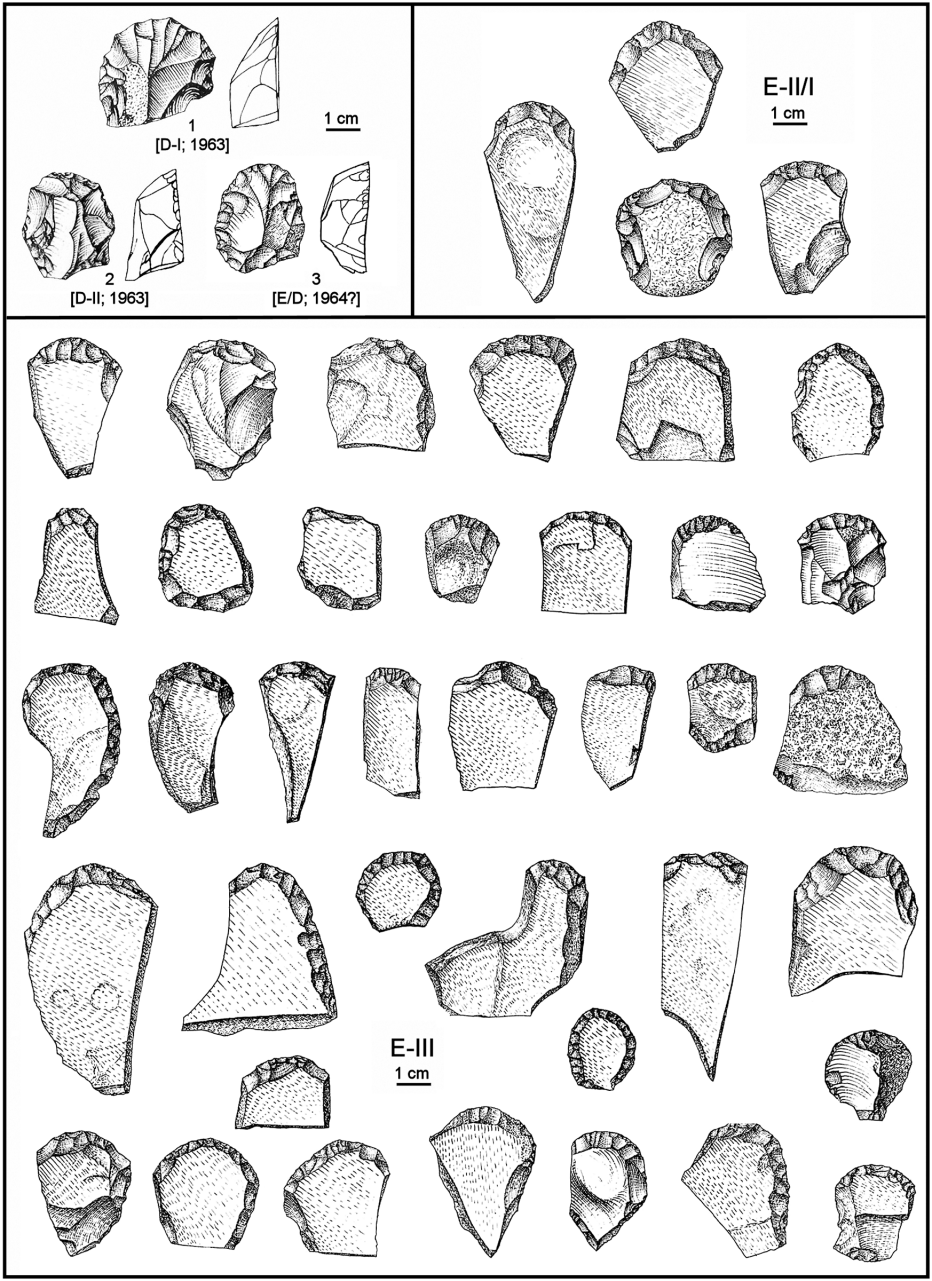
Uluzzian endscrapers vs. Aurignacian carinated scrapers/cores at Grotta del Cavallo. **Top left:** carinated items (in fact, cores for the extraction of blanks such as those of the bladelet tools in Fig 4) published as coming from layer D and level E/D; for each item, the level of provenience and year of discovery are indicated. **Top right and Bottom**: for comparison, the originally illustrated “endscrapers” from levels E-II/I and E-III, which differ in both blank type and mode of edge modification. Reproduced from refs. [19,21,23].

This conclusion also sheds light on items such as nos. 1–3 of Fig 5. Although the classification of no. 1 as a carinated scraper/core has been questioned [18] on the basis of it being less thick than implied by the representation of its cross-section in ref. [38], Palma di Cesnola had no doubts: this is the item that he selected as an example of the “several endscrapers of aurignacoid type, including some nosed and carinated ones, at times of remarkable workmanship” found in layer D [23] (p. 24). Where the classification of no. 3 is concerned, the excavator was equally adamant: “a beautiful frontal carinated endscraper opposite a *déjeté* carinated nose” [23] (p. 20). In fact, the key point concerning these three items is the nature of the “retouch” seen in their “endscraper front.” The convergent and lamellar nature of the scars is fully consistent with their use as cores for the extraction of small, narrow blanks such as those of the Dufour bladelets illustrated in Fig 4; it stands in stark contrast with the edge modification typical of the E-III and E-II/I items classified as endscrapers by Palma di Cesnola. In order to make this point more clearly, Fig 5 also reproduces the drawings of E-III and E-II/I “endscrapers” provided in Palma di Cesnola’s monographic study of the site’s Uluzzian stone tools [21,23]. This comparison further illustrates how the distinct contrast in “retouch” is complemented by an equally distinct contrast in blank type. Palma di Cesnola’s layer E “endscrapers” are made on cortical blanks extracted from *lastrine* (locally available thin plaques of siliceous limestone) or from small flint pebbles. In contrast, the items featuring “fronts” with convergent/lamellar scars (Fig 5, nos. 1–3) are made on blanks whose thickness and dorsal aspect indicate extraction towards the latter stages of more complex reduction sequences; sequences that would have required larger, thicker blocks of raw-material.

Although unknown in the Uluzzian and typical of the Aurignacian, one must also bear in mind that carinated and nosed scrapers/cores are not exclusive to the latter. In southwestern Europe, they are rather common in assemblages dated to the transition between the Gravettian and the Solutrean, at the beginning of the Last Glacial Maximum (LGM) [44]. In Italy, they are also common in the Early Epigravettian—up to 43% of the endscraper tool category in the assemblage from layer 17 of Grotta Paglicci, for example [4] (p. 226). As items clearly indicative of the Early Epigravettian are present in the assemblages from layers D and B of Cavallo (see below), the possibility that nos. 1–3 of Fig 5 relate to this time period cannot be excluded.

Bearing in mind the potential presence of even later intrusions, close inspection of the illustrations, tables and descriptions of the 1963–64 material from layer D indicates, however, that most of it—not just the items in Figs 4 and 5—is Protoaurignacian or Aurignacian in origin. For instance, almost all of the “endscrapers” in levels E-III (N > 180) and E-II/I (N = 22), are made on *lastrine* (Fig 5); however, this raw-material is completely absent among the 15 true endscrapers from layer D. Where burins are concerned, Palma di Cesnola [21,23] states that they are entirely lacking in level E-III and represented by a single, questionable specimen in level E-II/I. However, in Fig 3 (nos. 11, 12) of ref. [19] he illustrates a couple *bona fide* burins from levels E-II/I and D-II that, while clearly extrinsic to the Uluzzian, would not be out of place in an Aurignacian context. Lunates, the emblematic Uluzzian tool, represent 11.1% of the retouched tools in level E-II/I but only 4.1% in level D-II and 0.8% (one object) in level D-I [23] (pp. 54–55). Finally, the substrate category is, in levels E-III and E-II/I, overwhelmingly dominated by sidescrapers made on *lastrine* that, judging from both the illustrations and our own observation of the Taranto collection, form a continuum with the “endscrapers” of our Fig 5 that are made on similar blanks; in contrast, the 49 “sidescrapers” from layer D correspond, for the most part, to continuously retouched or denticulated pieces, 89.8% of which are made on flint flakes instead of *lastrine*.

**Fig 6:**
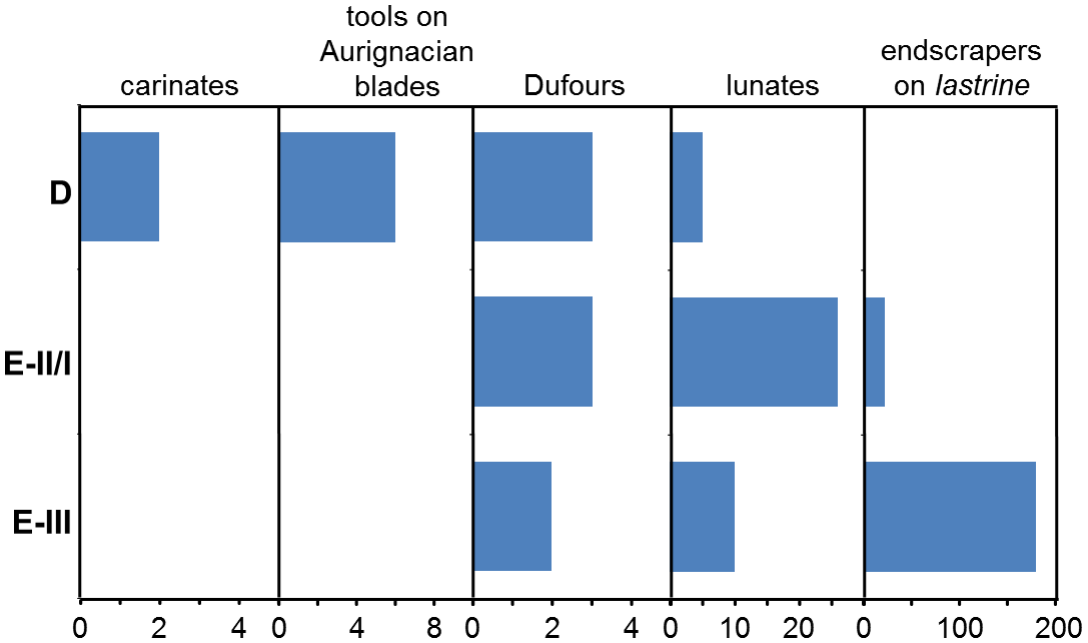
Vertical distribution of diagnostic lithics in layers D and E of Grotta del Cavallo. For carinated scrapers/cores, tools made on Aurignacian blades, and Dufour bladelets, only those illustrated in refs. [19,21,23] and whose typological classification is corroborated by the associated description have been counted. The numbers for lunates are drawn from p. 55 of ref. [23] and subsume the three items assigned to level E-II/I classified under the Laplacian categories Gm 2 (triangles) and Gm 6 (trapeze). Counts for “endscrapers” on *lastrine* (locally available thin plaques of siliceous limestone) are based on the text, figures and tables in refs. [21,23].

The vertical variation of these tool categories across the main subdivisions of Palma di Cesnola’s “Uluzzian” sequence is provided in Fig 6. Despite the overlap in microlithic types, it is clear that the layer D assemblage is distinct; it is not just that items of clear Aurignacian affinities such as those discussed above appear for the first time, but, more importantly, that the kinds of items defining the Uluzzian at the site—the lunates and the tools made on *lastrine*—all but disappear.

That the layer D assemblage is structurally distinct is further made apparent by consideration of the ornamental shell material. According to Palma di Cesnola [23: 36–37], the vertical variation observed in this material is as follows: level E-III contained only “three or four” *Dentalium* shells; level E-II/I also had *Dentalium*, dominating but in unspecified number, as well as a few shells of *Pectunculus* (= *Glycymeris*) and some, very small *Natica* and *Trochus* (= *Osilinus*) specimens; layer D, in contrast, yielded “very abundant” *Dentalium* tubes, “significant numbers” of, often perforated, *Cyclonassa* (= *Cyclope*), “less frequent” *Trochus*, *Pectunculus* and *Nassa* (= *Nassarius*), as well as “rare” *Cerithium* and *Columbella*, the latter also often perforated. Readers familiar with such material will have no difficulty in recognizing in the layer D assemblage a spectrum of ornamental shell taxa commonly found in the Protoaurignacian and the Aurignacian of Mediterranean Europe [45,46].

The evidence reviewed above begs the question of why did Palma di Cesnola maintain that these assemblages were homogeneously Uluzzian despite his correct identification of Aurignacian affinities among the material from layers D and E. The answer lies in that, as is clear from coeval papers [27], he viewed the Middle-to-Upper Paleolithic transition in a Laplacian theoretical context. According to this perspective, the first Upper Paleolithic of Europe was a syncretic entity, the Aurignaco-Perigordian “*synthétotype*,” out of which two industrial phyla—Aurignacian and Perigordian—would eventually develop; in Palma di Cesnola’s application to the Adriatic coast of Italy, this translated into the transition being conceptualized as a continuum whereby the Uluzzian—which he saw as a Mediterranean facies of the Lower Perigordian, a.k.a. Châtelperronian [21] (p. 33)—bridged the Mousterian with the Protoaurignacian and the latter was the result of a gradual development of technological aspects already latent or incipient in the preceding phase. Yet, the fact that the Aurignacian lithics could not have been produced in the framework of the Uluzzian technical system implies that the layer D assemblage cannot be seen as standing for a “transitional” industrial entity.

We conclude that the composition of the layer D assemblage only admits two alternatives: (a) that layer D is an undifferentiated palimpsest mixing remains of Uluzzian, Protoaurignacian and Aurignacian occupations; or (b) that, primarily, layer D is a Protoaurignacian deposit that also contains Uluzzian and Aurignacian items in inherited or derived position. The latter is, in our opinion, the parsimonious hypothesis, and is further supported by the radiocarbon dating results and the evidence concerning the post-depositional disturbance of this part of the Cavallo sequence, to which we now turn.

### Post-depositional disturbance and assemblage integrity

#### Insights from published information

**Fig 7:**
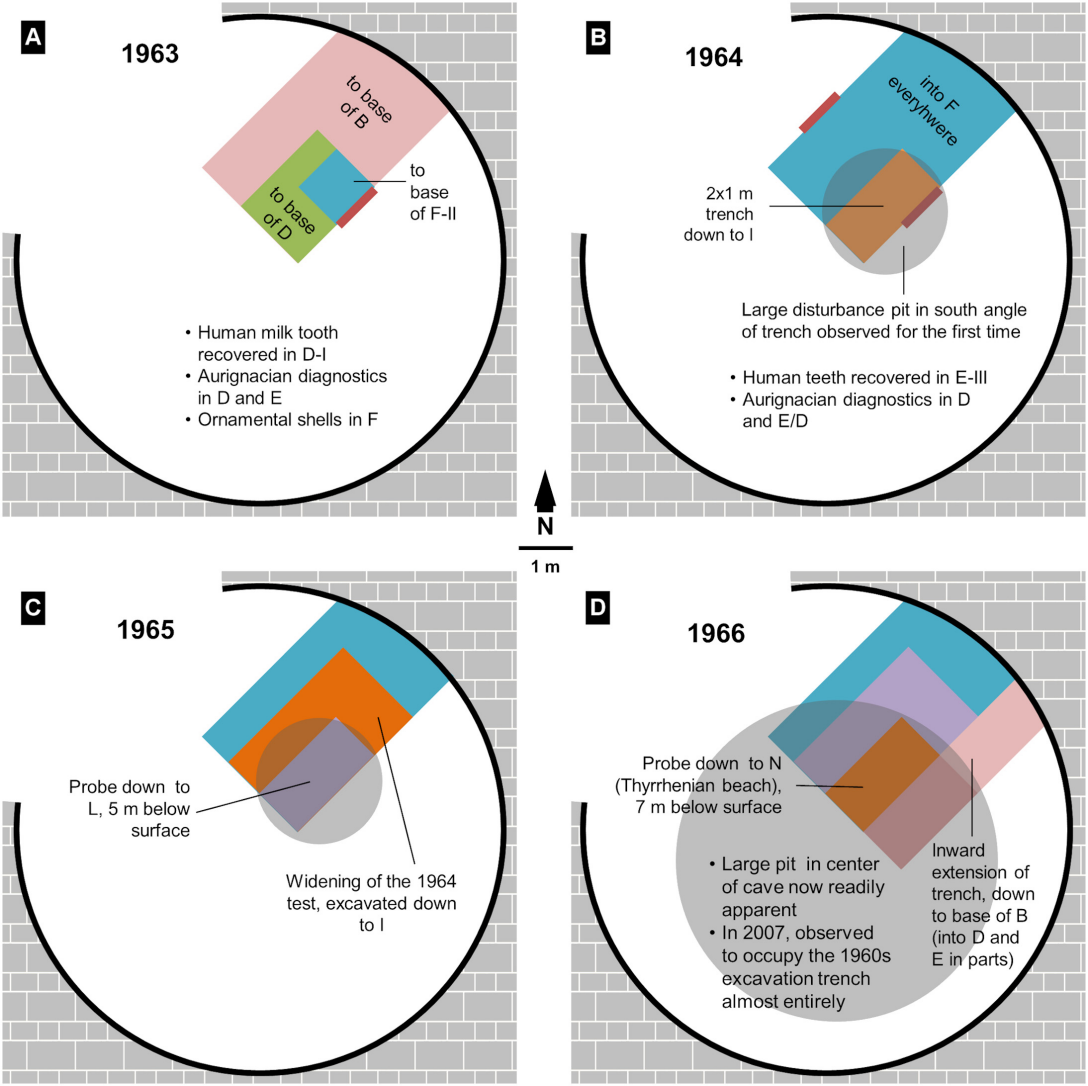
The annual progress of the Grotta del Cavallo 1960s excavations. A. 1963; B. 1964; C. 1965; D. 1966. Schematic plan with the approximate position, size and stepping of the excavation trench (colored rectangles), and of the stratigraphic cross-sections reproduced in Fig 3 (red bars), as inferred from the excavator’s descriptions; the stratigraphic depth reached and the significant finds made in each year are indicated. This reconstruction is provided as a graphical aid to the in-text discussion and should not be taken to represent an accurate rendering of the actual layout of site and trenches.

In order to properly assess alternative explanations for the nature of layer D, one would need adequate information on the site’s topography, stratigraphy and formation processes. However, to date, no plan of the cave has been published, no geological study of the sequence exists, and illustrations of its stratigraphy have been restricted to schematic renderings (Fig 3); in a couple of instances [12,32], limited photographic documentation of profile details has also been supplied. Based on the descriptions of the excavation work that appeared in print [19–24,31–33], it is nonetheless possible to propose a tentative reconstruction of the excavation’s progress (Fig 7) and of the nature of the stratigraphic problems encountered along the way.

The excavator describes the site as a cave with an overall circular outline, a diameter of ca. 9 m, and a ca. 5 m-wide entrance open to the NW. He states that the initial 1961 test trench was opened adjacent to the NE wall and that, in 1963, this test was extended to form a ca. 2.5–3 m-wide, 3.5 m-long trench in which layer B was completely excavated, layer D was excavated over a smaller area, and layer E was recognized in an area of ca. 1 m^2^ taken down to level F-II of layer F.

The schematic stratigraphy illustrating the 1963 excavation report (Fig 3) is said to represent “the most complete and most intact stratigraphic series as apparent in one of the trench’s sectors” [19] (p. 44); according to the caption, it was observed in the SE wall, and it details the subdivisions observed in layer C. With respect to this layer, it is also stated that “the C-I and C-II volcanic sands were especially visible along the central part of the cave, while in the proximity of the NE wall one passed directly from B-II to D-I and only isolated, partly ‘digested’ remnants of the red and grey aeolian sediment remained” [19] (45–46). From the representation in Fig 3 of layers E and F and of a thick and subdivided layer C we can therefore conclude that (a) the 1 m^2^ test that reached layer F was located towards the corner of the trench closest to the center of the cave, and (b) assuming that the work proceeded in the stepped manner characteristic of stratigraphic excavations, the part of the trench where the base of layer D was reached would have been located around that 1 m^2^ test, i.e., towards the central part of the cave as well.

The terms of the 1963 report already make it clear that descriptions of the Cavallo sequence whereby layer D would be “sealed” by the volcanic ash layer C [12,18] grossly oversimplify the true stratigraphic situation. At that time, however, the differences between different parts of the trench seem to have been interpreted as lateral variation. Post-depositional disturbance is only reported for the upper part of the sequence, consisting namely of “fox burrows and (…) pits containing remains from the Neolithic and more recent periods that penetrated more or less deeply in the underlying Pleistocene deposit” [19] (p. 43). Since they are clearly described, it can be assumed that the finds made in such disturbance features were excluded from the 1963 report’s discussion of the material culture of layers B to F. Even so, where layer D is concerned, the excavator made an explicit warning (our emphasis): “of the three spits into which it was subdivided, the deeper two (D-II) were different from the overlying one (D-I) in that the latter, besides presenting distinctive characteristics (…), **was also susceptible to some contamination**” because “in some sectors of the excavation not only was the sandy level C lacking (…) but the horizon D-I itself presented, especially towards the NE wall, a somewhat darker tonality that made it less easily distinguishable from the lower part of B-II” [19] (p. 52–53).

In 1964, the entire trench was brought down to the upper part of the Mousterian deposit, and the test unit that in 1963 had reached layer F was enlarged to a 2×1 m excavation that reached layer I, ca. 4 m below surface (Fig 7). Since layer B had been completely excavated the year before, the 1964 season mostly concerned layers D and E. Their separation was now seen to be significantly less clear, and the decision was therefore made to isolate their interface as a separate level, named E/D and corresponding to spit E1 [23] (p. 54–55). It was also at this time that a major disturbance feature was first noticed: “in the south corner of the trench,” layer C “was cut by a pit of undetermined age that probably extends across the entire central area of the cave,” one whose lower reaches affected, toward the trench’s SW wall, “a conspicuous portion of layers D and E (…) down to within a few centimeters of the Mousterian red soil (F)” [20] (p. 25). This observation probably explains why the stratigraphic section chosen to illustrate this part of the sequence in the 1964 report (Fig 3) is referred to the trench wall opposite that used for the same purpose in 1963, i.e., why it concerns the NW rather the SE wall. Since it shows the separation between C-I and C-II (the former now further subdivided into C-Ia and C-Ib), and we know from the 1963 report that such a separation was particularly apparent towards the center of the cave, the 1964 drawing must summarize observations made near the western corner of the NW wall, as inferred in our Fig 7.

The key corollary of these inferences is that the disturbance feature first recognized in 1964 affected an area of the trench where layers D and E had been excavated the year before. In other words, at the time of excavation, layers D and E of the 1963 trench were erroneously considered to be intact; in fact, they were significantly disturbed by the pit feature identified the following year, which, towards the center of the cave (i.e., in that restricted part of the trench where, in 1963, layers D and E had been excavated), traversed the site’s Upper Paleolithic levels down to their contact with the underlying Middle Paleolithic.

The 1965 field season focused on probing the basal Mousterian deposits, so it was not until 1966 that the extensiveness of the pit could be confirmed. In the excavator’s own words, the widening of the excavation area inward from the SE wall of the 1963–64 trench “better highlighted the large pit (already observed in previous years) filled with a brown sandy soil similar to overlying deposits that penetrates deeply into the volcanic sands C and the Uluzzian levels D–E” [24] (p. 290).

In 1972, when discussing the stratigraphic context of a group of Romanellian engraved blocks found in both layer B and the pit feature, the excavator described the latter’s configuration as follows [26] (pp. 52–53):

“The most eye-catching disturbance is represented by a large pit open towards the center of the cave, one whose horizontal boundaries remain to be established, as our excavations have not yet been completed. The pit, which, at least in the area explored, presents a slight outward flare, with irregularly curved or jagged margins, beginning clearly only at the level of B-IIb, cuts the entire thickness of the volcanic sands corresponding to stratum C, penetrating deeply into the Castelperronian deposit, reaching its base in some points. The filling of this cavity is represented by a brown sandy terrain, very similar to that which characterizes level B-IIb.”

These observations were confirmed in the 1980s, once the damage caused by looters was cleaned and excavations, now undertaken by P. Gambassini for the Uluzzian levels [31], could resume. At this time, it became apparent that the pit “occupied almost the entire excavation trench” of the early 1960s, and was “affected by numerous, deep and articulated rodent burrows” [33] (p. 421).

**Fig 8:**
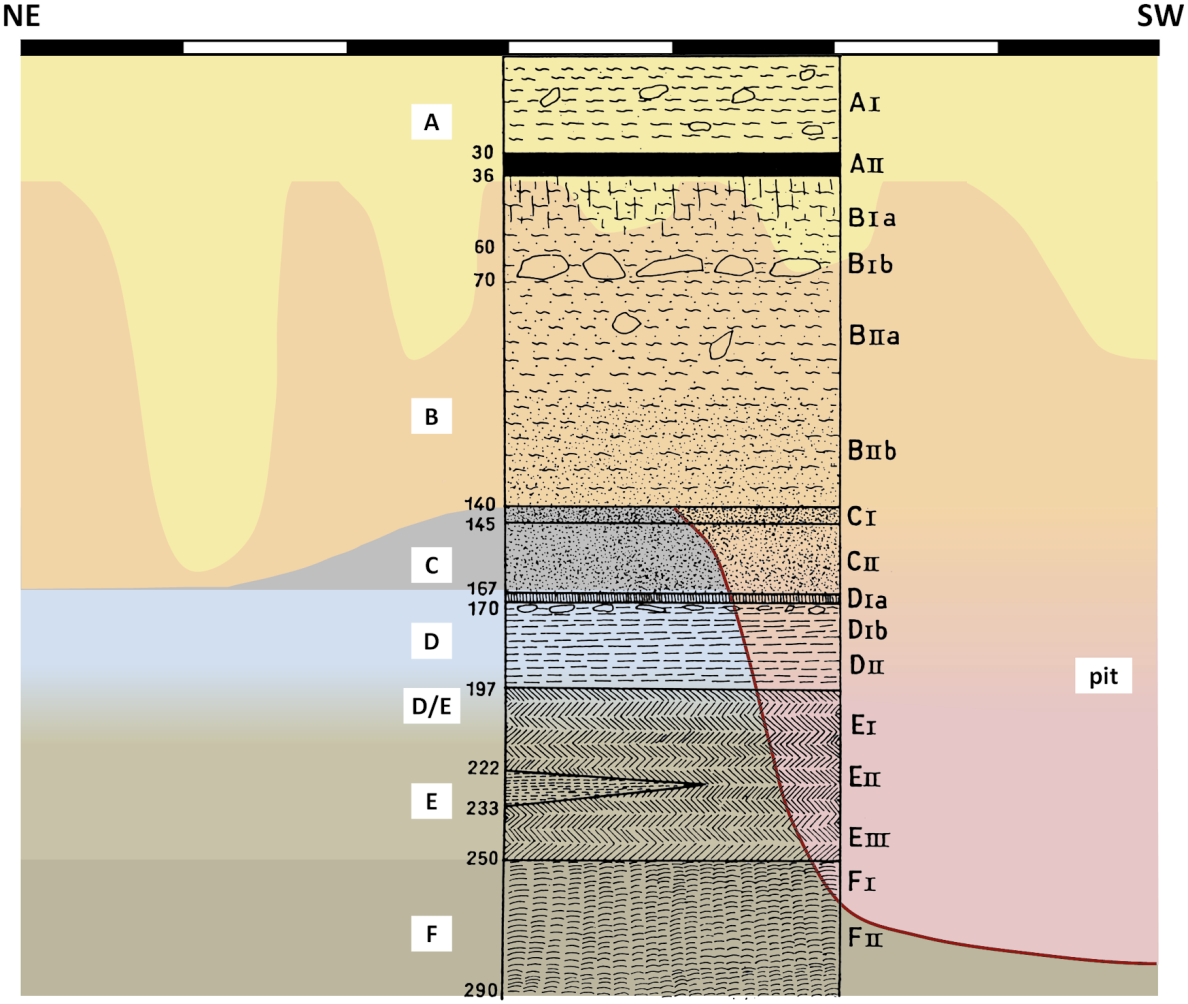
Stratigraphic configuration of the upper part of the Grotta del Cavallo deposit. Superimposition of the 1963 schematic profile on our reconstruction of the trench’s 3.5 m-long SE wall as it would have appeared, based on the excavator’s descriptions (see text), at the end of the 1964 field season. Elevations are in centimeters below surface.

The real stratigraphic configuration of the SE wall of the 1963–64 trench would therefore have been as schematized in Fig 8. Concerning the integrity of the assemblages from layers D and E of Cavallo, this configuration, combined with Palma di Cesnola’s account of the excavation’s progress, has the following implications:

- since the pit was identified in 1964, it can be inferred that, in the course of the 1966 season, its contents would have been excavated separately and, therefore, that finds made in 1966 and assigned at that time to either of those layers were made in broadly intact deposits;
- by the same token, the opposite case must be assumed for the 1963 material, as it comes entirely from an area of the site that was affected by the pit even though its existence went unrecognized at that time;
- the cultural-stratigraphic significance of the finds made in 1964 is open to question; this is because, even if the existence of the pit had been recognized prior to completion of that year’s excavation work, the corresponding finds would still come in part from areas excavated between the start of the season and the undetermined moment when the disturbance was first noticed; moreover, given that it was only after the 1966 and subsequent field seasons that the true extensiveness of the feature became apparent, it is clear that the pit’s boundaries were not (and indeed could not) have been accurately delimited in 1964.

In this context, maintaining that (a) the issues of stratigraphic integrity affecting the 1963–64 trench had limited impact, (b) the pit’s boundaries were precisely defined and its contents separately excavated from the very beginning and (c) layer D is truly Uluzzian [12,18], would be possible only if the items of unambiguous Aurignacian affinities from Cavallo had all been retrieved in the uppermost part of layer D, level D-I. Given the indistinctiveness of the boundary with layer B wherever the intervening volcanic ash (layer C) was lacking, level D-I was susceptible to contamination, as noted by the excavator; this circumstance would therefore suffice to explain how items originally deposited as part of a hitherto unrecognized Aurignacian occupation at the base of layer B ended up among the layer D assemblage, and vice-versa. Such a contamination, however, need not be very significant, and would in any case be of little consequence for the interpretation of layer E.

The problem, however, is limited neither to the B-II/D-I interface nor to the 1963 field season. Where stratigraphic provenience is concerned, this is shown by (a) the Dufour bladelets illustrated in our Fig 4, as two are from level E-III, three from level E-II/I and two from level D-II, and (b) two of the carinated scrapers/cores illustrated in our Fig 5, which come from levels D-II and E/D. Where year of excavation is concerned, this is illustrated by the ink-markings denoting the 1964 field season (see below) that, according to PG’s 1985–86 records, are borne by nos. 11–13 of Fig 4: 1U78B, 1U18B and 1U52B, respectively. We do not have information concerning the ink-markings of the five items labelled “1964?” in Figs 4 and 5, but we infer that such is their year of excavation from the comparison between total type counts given in the 1963 report and in the 1965–66 monographic study of the totality of the 1963–64 material: 198 and 212, respectively, for layer D; 217 and 969, respectively, for layer E [19,23]. The 1964 field season therefore contributed to the inventory with 766 additional items, of which 14 (5.5%) came from layer D and 752 (94.5%) from layer E. Even if we account for the possibility that the 1965–66 study relegated to “debitage” some material counted among the “tools” in 1963 (e.g., because “retouch” was reinterpreted as “damage” or “use-wear”), it is clear, given these percentages, that the items of major typological significance from layer E that were not mentioned in the 1963 report (namely, the Dufour bladelets with nos. 6–8 of our Fig 4) indeed are likely to come from 1964.

In addition, the 1963 report mentions shells of *Dentalium*, *Columbella* and *Cyclonassa* among the material recovered in Mousterian layer F [19] (p. 46); although present in layer D, the latter two taxa are absent from layer E [23] (pp. 36–37), and similar finds of ornamental shells were never again reported for layer F, be it in the 1964–66 excavations or in those conducted through the 1980s and 1990s [31]. In the case of *Cyclonassa*, one must also bear in mind that direct dating of a specimen assigned to this taxon and reported to come from level D-I of the 1963 excavations yielded an age of 19,685 ± 75 BP (OxA-21072) [12]. Taken together, this information makes it clear that the ornamental shell component from layer F of the 1963 field season is made up of items intruded from much higher up in the sequence. Their presence as far down as the Mousterian therefore corroborates the conclusions derived from the scant historical information discussed above, namely that (a) in the 1 m^2^ probe into layers E and F excavated in 1963, most sediment consisted of the reworked fill of the pit feature first identified in 1964, (b) the same applies at least to some extent to the parts of the trench excavated in 1964, (c) the mixed nature of such disturbed deposits is reflected in the composition of the “layer D” and “layer E” find horizons, and (d) inferences that rely on an assumption of homogeneity for the assemblages associated with any of the subdivisions of these stratigraphic units cannot be supported.

Needless to say, these conclusions have implications for the cultural assignment of all other finds made alongside the lithics and the shell ornaments. Namely, if both later and earlier material exists in Protoaurignacian layer D, and Protoaurignacian, Aurignacian and even later intrusions exist in Uluzzian layer E and Mousterian layer F, then it is clear that any human teeth reported to come from layer E cannot be assumed to be Uluzzian simply on the basis of the stratigraphic designation they came to be associated with.

#### Insights from the Taranto collection

The Taranto depot of Apulia’s Direction of Antiquities (*Soprintendenza per i Beni Archeologici della Puglia*) contains two boxes with finds from the 1963–64 Cavallo excavations. The lithics are ink-marked with codes and grouped in (a) waxed-paper bags bearing a yellow adhesive tag on which their stratigraphic provenience has been written, or (b) plastic bags containing a paper label with that stratigraphic information. The shell ornaments are not marked but their provenience is indicated on the associated labels. There are also bags containing pottery and miscellaneous bone and shellfish remains from the uppermost, Holocene levels of the sequence. In addition, each bag bears a penciled number (or that number is written on the paper label found inside) referring its contents to the corresponding catalogue record (*scheda*) of an inventory elaborated in the early 1970s by archeologist Mariantonia Gorgoglione. At the time of our analysis of the collection (June 2014), only the finds from layers B and F were available; the material from layers D and E once also kept in the Taranto depot had been transferred to the University of Siena in 2009 for study. However, we could examine a selection of lithics from these layers that had been mounted for display, in preparation for a future exhibition; their ink-marking, inventory number, and stratigraphic assignment are logged in the master catalogue of the National Archaeological Museum of Taranto.

M. Gorgoglione’s *schede* provide information on the composition of the Cavallo collection at the time it originally went into storage, more than four decades ago, while a list of the material recently moved back to Siena, supplemented with photographs, is kept in the Taranto depot. The comparison of these documents makes it apparent that important sets of finds made in 1963–64, ones that are explicitly referred to in Palma di Cesnola’s accounts (in some cases, even individually described or illustrated), must have remained in his possession or kept at the University of Siena ever since his excavations; in fact, they are not in storage at Taranto, do not appear in the 2009 list of transferred items, and are not mentioned in the early 1970s *schede*. Such is the case, namely, with (a) the diagnostic Aurignacian lithics discussed in preceding sections, (b) all the bone tools, and (c) the marine shell ornaments from the 1964 field season (the *schede* only list shell ornaments from 1963, and we could indeed relocate and record all those listed therein). This inference is corroborated by the fact that, when researching the Cavallo lithics in the mid-1980s, one of us (PG) had to examine part of the collection in Taranto and another part in Siena—and it was in the latter that all of the site’s Aurignacian diagnostics were observed.

In both collections (Siena and Taranto), the code used in the lithics’ ink-marking is of the type “U.#?” or “1U.#?,” where “#” stands for a number and “?” for one of three upper case Latin alphabet letters: A, B and G. From the comparison of item markings with the label of the bag they were in and the year of excavation given in the corresponding *scheda*, it can be concluded that the prefix “U” was used for the 1963 material and the prefix “1U” for the 1964 material. This conclusion is consistent with the fact that most “U” material is found in bags referred to layer B and most “1U” material is found in bags referred to layers D and E, in agreement with Palma di Cesnola’s account of the excavation’s progress. From our examination of the contents of the material from layer F, largely untouched and whose contents are an almost perfect match to the corresponding descriptions in M. Gorgoglione’s early 1970s *schede*, it is clear that the second part of the code concerns the item’s spatial and stratigraphic provenience, as the entire contents of such bags are marked in the exact same manner. For instance, bag No. 386, labeled “Must” and “F,” contains 120 flakes, sidescrapers and fragments thereof, all made on *lastrine* and all ink-marked “1U53A,” while bag No. 396, labeled “F-II/I,” contains 52 such items all ink-marked “1U57A” (S1 Appendix). However, we were unable to find in the Taranto archives any table detailing the correspondence of those codes with the actual grid units and excavation spits used in the field.

Although insufficient for a full analysis, additional information on the stratigraphic integrity of the Cavallo assemblages can still be gleaned from this body of data. A potential issue here is the extent to which the Taranto collection is exempt from curatorial mix-ups generated by decades of scholarly examination of the material. For instance, we identified apparent stratigraphic associations between items with clear late Upper Paleolithic affinities and items that are characteristically Middle Paleolithic. While some document post-depositional disturbance, others are clearly a result of such mix-ups. The latter is the case, for instance, with bag No. 85, which we found together with other bags of Mousterian lithics and itself bore a yellow adhesive label with “Must” written on. It contained five blades/bladelets extracted from prismatic cores of high quality flint and 14 other bladelets and lamellar debris extracted from either prismatic or bipolar cores of similar raw-material, all of them with a “U” marking denoting the 1963 field season; yet, according to the corresponding *scheda*, bag No. 85 should have contained two scrapers from level F-II/I of the 1964 excavations. We have identified other instances of this problem but, overall, they affect a small percentage of the collection available for analysis in Taranto: 63 out of 703 bagged items for which we had *scheda* information, i.e., less than 10%. Excluding these items as well as others for which some degree of uncertainty existed (e.g., because of information missing in the *schede*), correspondence between the contents and labels of the bags and the descriptions and stratigraphic assignations of the early 1970s inventory could be established for 671 (587 stone tools and 84 pieces of ornamental mollusk shell) out of the 787 items assigned to layers B, E and F that we analyzed in June 2014 (i.e., 85%; Table 1). This result indicates that we can confidently use the stratigraphic information written on the bags containing these 671 items as an accurate reflection of where in the Cavallo stratigraphic sequence Palma di Cesnola placed them in 1964–65, when he wrote up his monographic description of the 1963–64 material [21,23].

**Table 1:** Reliability of the stratigraphic assignment indicated by bag labels for the Cavallo items curated at the Taranto depot as of June 2014.

BAG/*SCHEDA* UNIT OF ASSIGNMENT	MARKED LITHICS	UNMARKED LITHICS	LABELED SHELL (a)	TOTAL	RELIABLY PROVENANCED
Uncertain (b)	65	2	–	67	–
Layer B (total)	222	41	36	299	258
B-I	51	3	5	59	56
B-IIa	59	3	17	79	76
B-IIb	112	35	14	161	126
Layer D (total)	12	2	45	59	57
D-I	–	–	7	7	7
D-II	–	–	38	38	38
D	12	2	–	14	12
Layer E (total)	66	2	–	68	66
E-II/I	36	1	–	37	36
E-III	30	1	–	31	30
Layer F (total)	287	4	3	294	290
F-II/I	1	–	3	4	4
F-III	1	–	–	1	1
F	285	4	–	289	285
TOTAL	652	51	84	787	671

^(a)^ Counts mollusk shell ornaments with associated paper labels and found in bags whose *scheda* description matched the actual bag contents.

^(b)^ Includes all instances of evident or potential storage mix-ups (N = 63) plus odd instances of missing *scheda* information.

Based on the above data and reasoning, we cross-checked the grid/spit code ink-marked on each of the 587 lithic artefacts reliably provenanced against their bag/*scheda* stratigraphic information (Table 2). We found that, despite making reference to a single grid/spit excavation unit, certain ink-marked codes appeared on items assigned to different levels—and this not only where adjacent stratigraphic units are concerned as, for instance, some are found in material assigned to level B-IIb as well as to level E-II/I, in theory separated by at least 60 cm (Fig 3). In order to corroborate that this pattern was not due to unidentified inventory errors or storage mix-ups generated by manipulation of the collection in recent years, we then undertook a similar cross-check for the items (total: 1295) whose ink-marked code was recorded in PG’s 1985–86 inventory of the Cavallo lithics (total: 1807). We found a similar scatter (Table 3). It is of particular significance that, in both sets of data, material from provenience unit U15G—clearly, a layer B code—is found among the stone tool assemblages assigned to layer D and to level E-II/I. Likewise, we observed in the Taranto depot that some material assigned to E-II/I had been ink-marked U79A—clearly a B-IIb code (Table 2).

**Table 2:** Singular provenience codes ink-marked on reliably provenanced Cavallo lithics in storage at Taranto that M. Gorgoglione’s 1970s *schede* and the Taranto Museum catalogue assign to two or more different levels of the sequence (as of June 2014; N = number of marked items assigned to the given stratigraphic unit).

CODE	B-II	D (a)	E-II/I (a)	E-III (a)
U15G (b)	1 (B-IIb)	2	1	–
U20G	–	1	1	–
U46A	12 (B-IIb)	–	1	–
U79A	12 (B-IIb)	–	2	–
1U20A	–	–	1	3
1U83B	–	5	1	–

^(a)^ Items mounted for display in the Taranto Museum.

^(b)^ The following U15G-marked items were excluded from the table because of significant discrepancy between the contents of the bags in which they were kept and the description and/or counts recorded in the associated *schede*: eight pieces of blade/bladelet debitage/debris on good quality flint found in level B-I bags pencil-labeled “318” (which contained 20 items and should only have 9) and “319” (which contained 16 items and should only have 6); two bladelets from Upper Paleolithic prismatic cores on good quality flint found in a layer F bag paper-labeled “85” (which contained 19 such items but should only have two scrapers). These ten items confirm nonetheless that U15G is the code for a field unit that contained both clearly Upper Paleolithic material on good quality flint and Uluzzian or Mousterian material made on limestone (namely, the two scrapers and one splintered piece observed among the items mounted for display and assigned by the museum catalogue to layer D and to level E-II/I).

**Table 3:** Singular provenience codes ink-marked on Cavallo lithics that P. Gioia’s 1985–86 inventory of Uluzzian stone tools and the Taranto Museum catalogue assign to two or more different levels of the sequence (N = number of marked items assigned to the given stratigraphic unit).

CODE	? (a)	D	E/D	E-II/I	E-III	TARANTO DEPOT (JUNE 2014)
U1G	2	9	–	–	–	one exhibit item: E-II/I
U2G	6	8	–	–	–	one exhibit item: E-II/I
U15G	28	2	–	3	–	see note (a) to Table 2
U20G	24	–	–	6	–	one exhibit item: D
U46A	–	–	–	1	–	twelve bag items (*scheda* # 337, 339, 345, 346, 352): B-IIb; one exhibit item: E-II/I
U59A	–	9	–	–	–	three bag items (*scheda* # 100): level B-I
U96A	–	1	–	–	–	one exhibit item: E-II/I
1U8A	–	1	–	35	–	five exhibit items: E-II/I
1U20A	–	–	1	–	64	one exhibit item: E-II/I; three exhibit items: E-III
1U23A	–	–	–	21	–	one exhibit item: E-III
1U29A	–	–	1	–	10	one exhibit item: E-III
1U48B	–	–	1	1	–	–
1U50B	–	–	–	26	–	one exhibit item: E-III
1U61B	–	–	2	–	77	five exhibit items: E-III
1U65A	–	1	–	–	1	–
1U83B	–	27	–	–	–	five exhibit items: D; one exhibit item: E-II/I

^(a)^ Includes missing/unknown as well as all entries of the type “D?,” “E-II/I?,” and “E-III?”

These data strongly suggest that, after the 1964 field season, once he realized the extent to which the excavated deposit had been affected by the pit at the center of the cave, Palma di Cesnola undertook a certain amount of post-hoc reassignment of finds to a “layer” different from that to which they had been referred at the time of excavation. This practice was not uncommon at the time, and it has been shown that F. Bordes proceeded to do just that with the carinated scrapers/cores originally found in the Late Gravettian of his late 1950s excavations at Laugerie-Haute Est; he inferred they represented contamination and consequently re-placed them in the site’s so-called “Aurignacian V” level [44].

Where Cavallo is concerned, it is impossible to assess whether Palma di Cesnola did the same in a systematic manner or only for those parts of the trench he considered most suspect, with the intention of correcting perceived distortions in the composition of the artefact assemblages caused by post-depositional disturbance. Whichever the case, the evidence reviewed above indicates that the published stone tool assemblages are organized in a stratigraphic order that, to a certain extent, is “reconstructed;” and, where the counts given for key typological categories are concerned, much the same can be inferred from the discrepancies between the 1963 preliminary report [19] and the 1965–66 monographic study [21,23] (Table 4). The increase in the numbers for layer E seen in the latter study is easy to understand as resulting from the addition of finds made in 1964. The decrease in the numbers for layer D (from 16 to 4 cf. Dufours, and from 8 to 4 geometric microliths, cf. lunates), however, is harder to explain. Conceivably, it could stand (a) in the case of the Dufours, for the elimination of items whose modification was considered marginal retouch in 1963 and edge damage or use-wear in 1965–66, and (b) in the case of the lunates, for reclassification of some as backed or backed and truncated elements (types Ld2, Dt3–4 and Dt7–8), of which eight are counted in 1965–66 contra only two in 1963.

**Table 4:** Typological counts given by Palma di Cesnola in 1963 and 1965–66 for the Laplacian types in which he conceivably could have subsumed Dufour bladelets and lunates.

TYPOLOGICAL GROUPS	D-I	D-II	E-II/I (a)	E-III	TOTAL
**1963 preliminary report**					
Pd1	–	1	–	–	1
Ld1	3	9	5	–	17
*Punte*	3	–	–	–	3
TOTAL cf. Dufour bladelets (b)	6	10	5	–	21
Gm	1	7	–	–	8
Pd2&Pd4	–	5	7	–	12
Ld2	1	1	2	–	4
Dt3-4, Dt7-8	–	–	3	–	3
TOTAL cf. lunates (c)	2	13	12	–	27
**1965 monographic study of the 1963–64 lithics**					
Pd1	–	–	1	2	3
Ld1	3	1	10	3	17
TOTAL cf. Dufour bladelets (b)	3	1	11	5	20
Gm	1	4	51	10	66
Pd2&Pd4	1	2	28	4	35
Ld2	2	3	18	9	32
Dt3-4, Dt7-8	1	2	7	2	12
TOTAL cf. lunates (c)	5	11	104	25	145

^(a)^ Includes level E/D, corresponding to spit E1, which was considered part of E-II/I in 1963 and counted separately in 1965–66;

^(b)^ Laplacian microlithic types defined by the application of marginal retouch/backing, direct, inverse or alternate;

^(c)^ Laplacian types Pd2 and Pd4 are included given Palma di Cesnola’s classification of many lunates as Châtelperron points, especially in the 1963 report.

As the 1963 classification was carried out in collaboration with G. Laplace himself [19] (p. 56), we find it more likely, however, that these discrepancies reflect the post-hoc reassignment to level E-II/I of material from layer D. The 21 lunates of the Taranto Antiquities depot mounted for museum display in order to represent the contents of level E-II/I provide clear evidence of the practice, as three bear provenience codes that clearly refer them to layer D: U1G, U2G and 1U83B (Tables 2 and 3). In our view, there can be little doubt, therefore, that, in the wake of his 1964 recognition of (a) the diffuse nature of the boundary between layers D and E and (b) the major disturbance feature first identified that year, Palma di Cesnola did conduct a certain amount of post-hoc correction of the unit of provenience recorded in the field for items that he interpreted as misplaced (by excavation error) or displaced (by disturbance).

Additional support for these conclusions comes from the fact that we could find no ornamental shell material labeled as coming from layer F, and none is mentioned among the ornamental shells from the 1963 field season described in M. Gorgoglione’s *schede*; according to the published report, however, shells of *Dentalium*, *Columbella* and *Cyclonassa* would have been found at that time in the Mousterian levels [19] (p. 46). Therefore, even though he never stated it in print, it is quite possible that Palma di Cesnola eventually recognized the intrusive nature of this material, or the disturbed nature of the deposits from which it had been excavated, and, accordingly, proceeded to re-assign those finds to a different stratigraphic unit. This could also explain why the only three shells referred to as coming from layer F in the Taranto collection—a tiny, unperforated *Glycymeris* shell and two small fragments that are probably fossil and a natural component of the deposit (Table 5)—were found in a bag (No. 387) that (a) inside, matching the corresponding *scheda*’s description, contained a paper label with “F-II/I 1963” written on it, but (b) bore the “E-II/I” mention on the exterior yellow adhesive label. It is also conceivable that the 1963 ornamental shells from layer F have since been lost. However, given that the excavator never again mentioned them, we find it rather more likely that their apparent disappearance is simply another instance of post-hoc stratigraphic reassignment of significant finds (especially, but not exclusively, those from the 1963 season). Put another way, those “layer F” ornaments most likely still exist in the collection, but may now be labeled as coming from layers B, D or E, instead of F.

**Table 5:** Non-food mollusk shell finds from the Pleistocene deposits of the 1963 excavations at Grotta del Cavallo in storage at the Taranto depot of Apulia’s Direction of Antiquities (a).

TAXON	B-I	B-IIa	B-IIb	D-I	D-II	F-II/I	TOTAL
*Aporrhais* (perforated)	–	–	–	–	1	–	1
*Cerithium* (perforated)	–	–	–	–	1	–	1
*Columbella* (perforated)	–	4	–	–	–	–	4
*Cyclonassa* (perforated) (b)	–	11	13	6	8	–	38
*Dentalium* (perforated) (c)	–	–	–	–	22	–	22
*Gibbula* (?) (perforated) (d)	–	–	–	1	–	–	1
*Pectunculus* (large, unperforated)	–	–	–	–	2	–	2
*Pectunculus* (rolled “boomerangs”) (unperforated)	5	–	–	–	–	–	5
*Pectunculus* (small, rolled) (unperforated)	–	2	1	–	–	–	3
*Pectunculus* (very small, unperforated)	–	–	–	–	–	1	1
*Nassa* (perforated)	–	–	–	–	1	–	1
*Nassa* (unperforated)	–	–	–	–	1	–	1
*Natica* (unperforated)	–	–	–	–	1	–	1
Fossil (?) shell fragments	–	–	–	–	–	2	2
TOTAL	5	17	14	8	37	3	84

^(a)^ Genus names as used by the excavator and as entered in the collection’s inventory kept at the Taranto head office of Apulia’s Direction of Antiquities;

^(b)^ Includes two specimens, one from level D-I (*scheda* No. 355) and another from level D-II (*scheda* No. 353), removed for radiocarbon dating (according to a hand-written addition to the *scheda*, on March 9, 2009 and by or care of T. Higham);

^(c)^ Includes one specimen from level D-II (*scheda* No. 353) removed for radiocarbon dating (according to a hand-written addition to the *scheda*, on March 9, 2009 and by or care of T. Higham);

^(d)^ Classified as *Cyclonassa* in the Taranto inventory (*scheda* No. 355).

There are also indications that a similar procedure was adopted for significant finds made in areas definitely recognized (eventually, if not at the actual time of excavation) as affected by post-depositional disturbance. The fact that some such items were re-placed in the assemblage of the “layer” perceived to be the “correct” one on the basis of their typological features is illustrated by the largest (no. 12) of the retouched blades illustrated in Fig 4. This object is not mentioned in the 1963 report and appears for the first time in the monographic presentation of the Uluzzian assemblages written after the 1964 field season [21,23]. Since the Uluzzian deposit was apparently not excavated in 1965 (see above), the year of discovery of this blade tool is likely to be 1964, i.e., the year when the pit affecting the south angle of the trench was first recognized. This inference is consistent with the “1U” part of the object’s “1U18B” ink-marking. Accepting the argument that any finds made in areas affected by disturbance features would have been excluded from the presentation of the material defining the different cultural horizons [18], one would be led to believe that the retouched blade in question came from intact areas of the stratigraphic unit to which it is provenanced. Yet, we know that such is not the case. The reason being that, although assigned by Palma di Cesnola to layer D, this item was found inside a “*buco*” (hole)—as one of us (PG) previously stated [37] (p. 248), based on information received from the excavator himself.

These examples suggest that, even after the major pit in the central part of the cave was identified, the excavator downplayed the impact of post-depositional disturbance and boundary indistinctiveness on the composition of the assemblages retrieved from layers B to F. Instead of screening the material to retrospectively detect potential contamination and remove from analysis any and all items coming from affected areas, Palma di Cesnola seems to have attempted to correct perceived “anomalies” via the reassignment of at least some significant “out of place” items to the stratigraphic unit from which he thought they would have originated. This inference is further strengthened by the fact that provenience categories designated “disturbed” or “reworked” exist neither among the bag labels of the 1963–64 material kept in Taranto nor in the associated *schede* of the early 1970s inventory.

The number of singular provenience codes that can be found on artefacts assigned to two or more layers is, however, limited (N = 17); i.e., 12% of the combined total of 143 such codes known to us (77 from direct observation of Taranto depot items, the additional 66 from entries in PG’s 1985–86 inventory). Clearly, this figure is a minimum number. Even so, it suggests that the practice of post-hoc stratigraphic reassignment was targeted and affects neither the basic structure of the sequence’s vertical distribution patterns nor the reality of the Mousterian-Uluzzian-Protoaurignacian/Aurignacian succession—although it may have contributed to enhance contrasts in the composition of published lithic assemblages, namely those between layers D and E made apparent in Fig 6. Where key individual finds are concerned, however, this stratigraphic reassignment practice serves to add another layer of uncertainty to the issues of association raised by the problems of post-depositional disturbance and boundary indistinctiveness already discussed.

In conclusion: (a) the stratigraphic definition of the artefact assemblages from Cavallo includes a non-negligible amount of post-hoc reconstruction, especially where the 1963 material is concerned; (b) those artefact assemblages contain material derived from both intact and disturbed areas of the deposits. Needless to say, if such is the case with the stone tools and the shell ornaments, then we cannot exclude that such is also the case with the samples used for radiocarbon dating, or with the human fossils. Their published stratigraphic positions, cultural associations, and, thus, significance, must be assessed with this caveat in mind.

### Layer D: formation, definition and age

**Fig 9:**
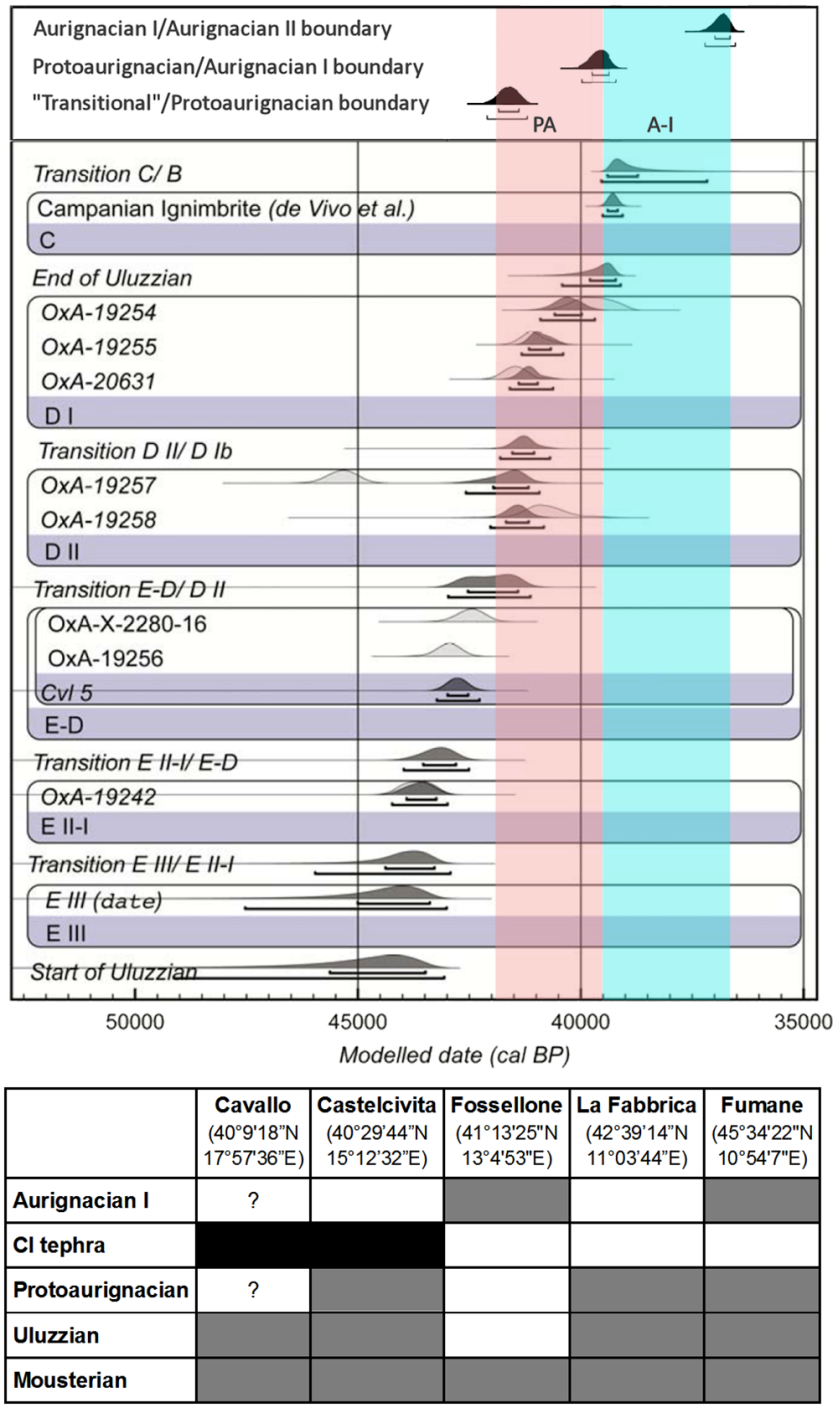
Uluzzian chronostratigraphy. **Bottom:** position of the Uluzzian relative to bracketing technocomplexes at key multi-component stratified sites in Italy; the grey-shaded and white cases denote presence or absence, respectively, of the corresponding stratigraphic units; the CI (Campanian Ignimbrite) tephra is not found in Italian sites north of the Phlaegrean Fields explosion locality (40°49’37.2” N, 14°8’20.4” E). **Top:** the boundaries of the Protoaurignacian (PA) and the Aurignacian I (A-I), as modeled in ref. [14], superimposed on the chronology of the Uluzzian at Cavallo proposed in ref. [12].

Excluding the much younger *Cyclonassa* radiocarbon age mentioned above and a stratigraphically anomalous age obtained on an undetermined bivalve fragment that could represent an inherited component of the deposit [18], the corpus of radiocarbon ages for layer D consists of four results obtained on marine shell [12]: three are *Dentalium* tubes; the other is a fragment tentatively identified as *Nuculana*. A Bayesian age model derived from these four ages places the accumulation of layer D in the time interval of ca. 39.5–41.5 ka cal BP (thousands of calendar years before present) [12]. Since (a) in Italy, the Uluzzian precedes the Protoaurignacian in both the North and the South, (b) such precedence is corroborated by radiocarbon ages obtained for both technocomplexes at Fumane (Verona) and Castelcivita (Salerno) [17], and (c) by 41.2 ka cal BP, the Protoaurignacian was already in existence in France, Italy and northern Spain [14], assigning layer D to the Uluzzian is in evident contradiction to chronostratigraphic patterns (Fig 9).

Given the evidence for post-depositional disturbance reviewed above, exploring possible causes for this contradiction requires that we first look at the provenience of the samples used in this dating project. Two are said to come from the 1960s phase of work—the already mentioned *Cyclonassa* from D-I and a *Dentalium* from D-II. The other six—four from layer D (two *Dentalium* plus the bivalve fragment and the possible *Nuculana*), one from level E/D, and one from level E-II/I (both *Dentalium* tubes)—are said to come from Uluzzian remnants excavated between 1978 and 1984 ([12] Tables S1,S5]. Specifically, we are told that the latter six “were recovered during rescue excavations in subsequent years and follow different assignation system,” and that “the correlation of the two series is based on field documentation.”

However, no documents or archival records have been provided in support of the “1978–84” provenience, and the table of equivalence between “Layers (1963–66)” and “Spits (1978–84)” given in ref. [12] is simply Palma di Cesnola’s 1966 table of correspondence between the analytical subdivisions (e.g., levels D-I, D-II, E-III) of his major stratigraphic units (e.g. layers D, E) and the 1963–64 spits (e.g., D1, D2, E1) that he actually used in the field [23] (pp. 54–55)—i.e., the “different assignation system” that would have been used in 1978–84 is nowhere reflected in the proposed correlation. In addition, we know that (a) the 1964 shell ornaments, never transferred to Taranto (see above), must have remained in Siena, where they would have been kept alongside any such material subsequently recovered and where the samples claimed to come from 1978–84 were indeed taken [12], (b) layers B, D and E were not excavated in 1965, (c) no ornamental shells are reported from the limited portion of layers D and E excavated in 1966 [24] (p. 290], and (d) the remnants excavated at a later time were primarily of levels E-III and E-II/I [30] (pp. 203–204) whereas the samples claimed to come from 1978–84 include material from layer D (spits D1, D2 and D3).

Therefore, we need to consider the possibility that at least some, if not all of the six “1978–84” shells are in fact from 1964. Were that to be the case, then those assigned to layer E might be argued to provide a dating context for the human teeth with the same provenance. However, the issues of post-depositional disturbance affecting the 1963–64 trench imply that the interpretation of the dates cannot be based on their putative stratigraphic position; in such a context, their ages can only gain meaning against an external frame of reference—the regional chronostratigraphic sequence. Put another way, assuming that the results are accurate, four distinct phases of occupation would be indicated by the Cavallo samples: Mousterian, ca. 42.4 ka ^14^C BP (thousands of radiocarbon years before present); Uluzzian, ca. 40 ka ^14^C BP; Aurignacian I and Protoaurignacian, ca. 36.5–34.5 ka ^14^C BP; and Early Epigravettian, ca. 20 ka ^14^C BP.

Conversely, if the “1978–84” samples indeed come from a later period of work carried out on intact areas of the deposit, they can then be taken, under the same assumptions and following ref. [18]’s reasons to exclude the bivalve fragment, to provide solid evidence for human occupation of Cavallo in two moments, ca. 40 ka ^14^C BP and ca. 36.5–34.5 ka ^14^C BP. The artefact assemblages defining such moments in terms of cultural-stratigraphic units would have to be, however, not the assemblages coming from the post-depositionally disturbed “layer D” and “layer E” of the 1963–64 trench but those collected alongside the samples in such intact “layer D” and “layer E” deposits, which remain unpublished. Therefore, not much changes for the significance of the samples if they were indeed obtained in 1978–84 and in intact deposits; in the absence of an immediate context, it is also the case that the results obtained for them can only gain meaning via post-hoc assignment, in this instance to the technocomplexes represented in the 1963–64 collection whose regional chronology they fit—Uluzzian, ca. 40 ka ^14^C BP, for the “1978–84/layer E” results, and Protoaurignacian and Aurignacian I, ca.36.5–34.5 ka ^14^C BP, for the “1978–84/layer D” results.

Regardless of whether the dated samples came from disturbed or intact areas of the deposit, the radiocarbon evidence therefore corroborates the presence in the Cavallo assemblages of Protoaurignacian- or Aurignacian-aged material—not just among the lithics but also among their ornamental shell component [16]. Against this conclusion, it has been argued that the lithic artefacts upon which it is based “would support an Early Aurignacian occupation (…) surely not a Proto-Aurignacian one that might be expected based on the radiocarbon data” [18]. The foundation of this argument is “the recent attribution of layer C, sealing the Uluzzian series E-III/D-I, to the Campanian Ignimbrite [CI],” which would contradict “the idea of an Early Aurignacian occupation being beneath it” [18] (p. 108). This objection is not valid, and this for three reasons.

Firstly, although the objection conceivably applies to the limited areas where an intact layer C existed, it obviously cannot apply to the site as a whole; given the extensiveness and depth of the disturbance affecting the sequence, the presence of Early Aurignacian (= Aurignacian I) and Early Epigravettian items in layer D of the 1963–64 trench documents post-depositional intrusion into a pre-CI sedimentary body, not that such items entered the archaeological record prior to the formation of the CI. Secondly, the Dufour bladelets illustrated in Fig 4 are consistent with the Protoaurignacian, which predates the CI; their presence in layers D and E would therefore be fully within stratigraphic expectations even if the layer C volcanic ash had truly “sealed” the underlying sequence from subsequent intrusions. Thirdly, the dated samples are but a part of the ornamental shell assemblage assigned to layer D; even though this layer’s *Dentalium* component could well be of Protoaurignacian age entirely, that cannot be taken to imply that such must be the case with all the lithics and all the other ornamental taxa found alongside. In fact, while the unmodeled results for the three *Dentalium* (36,260 ± 250 BP, OxA-19255; 36,780 ± 310 BP, OxA-20361; 36,000 ± 400 BP, OxA-19258) are statistically identical and within the Protoaurignacian range, the possible *Nuculana* yielded a statistically younger result (35,080 ± 230 BP; OxA-19254). This result overlaps the boundary between the Protoaurignacian and the Aurignacian I but, considering the marine reservoir effect, its 95.4% probability interval (39,753–38,641 cal BP) falls almost entirely in the latter’s range (Fig 9).

One must also bear in mind that ages obtained on ornamental shells from species of mollusks that were not harvested alive for alimentary consumption are maximum ages only; this is because the time elapsed between the death of the organism (the radiocarbon-dated event) and the time of collection and cultural use of its beached shell (the archeological event of interest) is unknown. In some instances, namely at Riparo Mochi [47], it has been shown that the lag between the age of the shell and its ornamental human use is short and falls within the uncertainty of dating results. However, the presumption that this is always the case is not valid, as has been shown in a number of other instances. An Italian case in point is Fumane, where ABOx-SC dating of Protoaurignacian charcoal samples from level A2 returned results between 34,180 ± 270 BP (OxA-19414) and 35,850 ± 310 BP (OxA-19584), while a *Glycymeris insubrica* ornament from overlying level D6 was dated to 37,100 ± 240 BP (OS-5872) [48,49]. For the Cavallo layer D ages that fall in the time range of the Protoaurignacian, it is easy to see how lags of comparable length would imply that such ages in fact stand for collection of the dated shells during the Aurignacian I and/or the Aurignacian II—and both are also plausible archeological contexts for the production and discard of, respectively, the blade tools and the Dufour bladelets illustrated in Fig 4.

The ages obtained for the radiocarbon-dated samples are therefore in agreement with the Protoaurignacian and Aurignacian technology and typology of the stone tools assigned to layer D. The fact that some Uluzzian diagnostics are also present therein (Fig 6) can be put down to post-depositional disturbance and/or the indistinctiveness of boundaries, while, conversely, such factors also explain the presence in layer E of material intruded from overlying layer D. The alternative interpretation is that (a) the ornamental shell dates are accurate and reflect the time of collection, (b) layer D is entirely Uluzzian, (c) thenceforth “the site appears unoccupied until the late Upper Palaeolithic period” but (d) the *Cyclonassa* shell is “obviously not related to the Uluzzian phase at the site” [18] (p. 109). This reading of the record is clearly inconsistent; an intact Uluzzian deposit cannot contain a non-Uluzzian shell bead that, in addition, dates to a time period during which the site is supposed to have been uninhabited.

**Fig 10:**
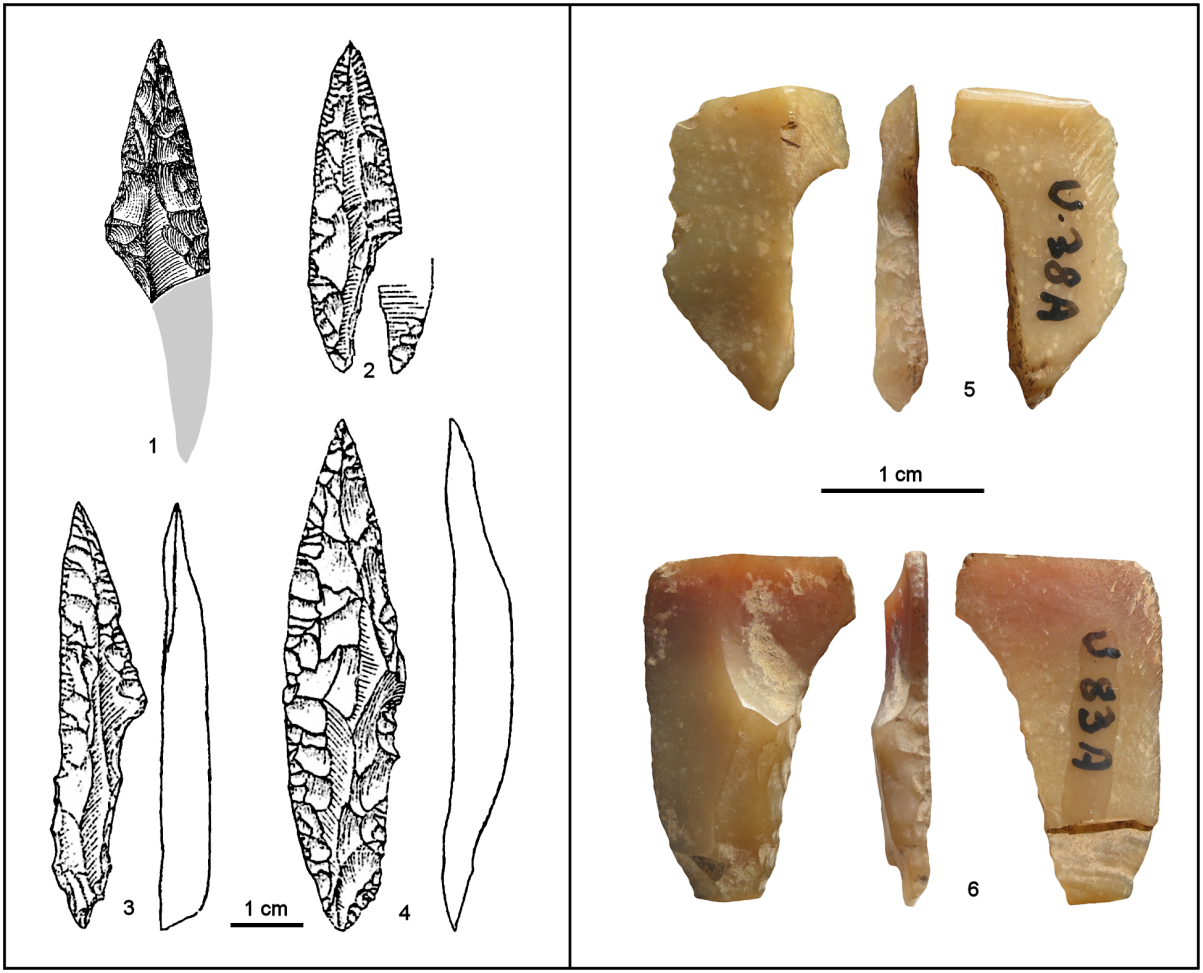
Early Epigravettian points from southern Italy. **Grotta del Cavallo:** 1, 5–6: distal (1) and proximal (5–6) fragments of shouldered points from level B-IIb of the 1963 excavations (the grey-shaded area in no. 1 stands for the missing tang; the excavator illustrated this item as no. 26 of Fig 8 of ref. [19] and described it as a “carenoïd point”). **Grotta Paglicci (level 18a):** 2–3: shouldered points; 4: *pointe à face plane* with flat, invasive retouch; reproduced from Fig 37 of ref. [4].

What the *Cyclonassa* age of ca. 20 ka ^14^C BP tells us is that layer D also includes artefacts from the LGM time range. Another indication that Cavallo was occupied at that time comes from an object illustrated by Palma di Cesnola among the level B-IIb stone tools [19] (reproduced in our Fig 10, no. 1). Although described as a Romanellian “carenoïd point,” this item corresponds in fact to the distal fragment of a shouldered point, as indicated by its size, its flat and invasive dorsal retouch, and its apparent impact fracture. We identified additional instances of the tool-type—two basal fragments (Fig 10, nos. 5–6)—in bag No. 338 of the Taranto collection; although never mentioned by Palma di Cesnola, these two objects are correctly identified in the corresponding *scheda* of the early 1970s inventory, which informs us that they come from level B-IIb of the 1963 field season. Shouldered points are characteristic of the Early Epigravettian as documented in southern Italy at e.g. Grotta Paglicci (Foggia; Fig 10, nos. 3–4), where levels 17E and 17B have been dated to the 20–18 ka ^14^C BP interval [4]—i.e., to the range of the *Cyclonassa* shell age from level D-I of Cavallo. Bearing in mind the excavator’s remarks on the indistinctiveness of the interface between B-IIb and D-I wherever the volcanic ash lens was missing, the fact that Early Epigravettian items were recovered on both sides of that interface should come as no surprise.

Additional corroboration that Cavallo was occupied during the Early Epigravettian (and, possibly, the Gravettian) is provided by the contents of other “B-II” bags found in the Taranto depot. These bags contain a number of robust microgravette points (or fragments thereof) that are more likely to belong in these periods than in the Romanellian, diagnostics of which (very small thumbnail scrapers, very narrow backed bladelets) are mostly to be found in bags with a “B-I” label. We speculate, therefore, that, instead of being homogeneously Romanellian, the ca. 1 m-thick layer B in fact spans a large portion of the Gravettian and Epigravettian time range. From the stratigraphic distribution of the *Cyclonassa* shells currently stored in the Taranto depot (Table 2) and their typological homogeneity (S2 Appendix), we further speculate that this type of shell ornament relates mostly, if not entirely, to such LGM occupations of the site. We can therefore derive from this evidence a strong presumption that the *Cyclonassa* specimens reported by the excavator to come from layers D and F represent post-depositional intrusion from layer B and that only the *Dentalium* tubes and the taxa with which these tubes were associated in layer D relate to the Protoaurignacian and/or Aurignacian occupations of Cavallo—as indeed indicated by the dating results.

When the site’s stratigraphic layout is duly considered, the existence of such Gravettian/Early Epigravettian material at this position in the sequence is entirely to be expected. Given that the last high sea stand of MIS (Marine Isotope Stage) 5 dates to ca. 81 ka [50], we can surmise that such is the age of the marine deposit forming the base of the Cavallo succession. Given that the discontinuous volcanic ash capping layer D is Campanian Ignimbrite, we also know that it dates to ca. 39.3 ka [51]. The deposits that accumulated in the 42,000 years separating these chronological benchmarks total ca. 7 m, and no significant hiatuses have been recorded in this part of the sequence; therefore, we can infer that, throughout, sedimentation at the site proceeded at a rate of ca. 17 cm/millennium. Thus, if the ca. 1 m-thick layer B were entirely Romanellian, i.e., if it had begun to accumulate no earlier than ca. 12,000 years ago, then (a) the rate of sedimentation at this time would have increased at least threefold to a minimum of ca. 50 cm/millennium, and (b) a 27,000 year-long hiatus would exist between layers C and B. In addition, as no evidence has ever been reported that the discontinuity at the interface between layers B and C might correspond to a major erosional scar, one would have to surmise that such a hiatus was caused by the accumulation of sediments in the cave having come to a halt; and, therefore, that the floor of the cave would have corresponded, throughout such a hiatus, to the surface of the pre-Romanellian deposit—i.e., to layer C, where preserved, or, in areas where run-off washed the volcanic ash away, to layer D.

We find the existence of a major sedimentation hiatus in a cave like Cavallo, with a large, wide-open, exposed entrance, to be unlikely. Nevertheless, if one accepts the notion, one then also has to face the additional implications deriving from Sarti et al.’s description of this part of the sequence [31] (p. 45; our emphasis): “The opportunity to resume research at Grotta del Cavallo came about at the end of the 1970s, when it could no longer be postponed, due to the affection of the archeological deposit by unauthorized digging; over a short period of time, the cave had become a kind of field school for dilettantes who, very quickly, due to **the pulverulent consistence of the Upper Paleolithic strata**, inflicted grave damage to the site.” In such a sedimentary context, the penetration subsurface (i.e., into layer D) of any artefacts abandoned at the cave in the framework of episodic human visits taking place during the 27 millennia-long hiatus putatively separating the CI event from the accumulation of the Romanellian sands would have been inevitable—as a result of normal soil formation processes, sheet wash and associated surface dynamics, and the burrowing activity of cave-dwelling animals. This would have been the case regardless of the consistency of the deposits; that they were “pulverulent” only means that the disturbance caused would per force be both more extensive and less easy to perceive than if the exposed sediments had been compact or cemented.

To sum up, our hypothesis is that, even though it eventually became “enriched” with components of a later age, the formation of the layer D deposit began during pre-CI, Protoaurignacian times. This hypothesis is fully consistent with both the stratigraphic configuration reported by the excavator for the undisturbed areas of Cavallo and the radiocarbon dating results obtained on ornamental shell samples. The opposite case [18] is that layer D formed in Uluzzian times and remained intact and pristine throughout an almost thirty millennia-long sedimentation hiatus during which it would have remained the cave’s exposed surface across those extensive parts of the site where layer C is missing; this latter case challenges observation and, in our opinion, amounts to a geological impossibility.

### Provenience of the human dental remains

**Fig 11:**
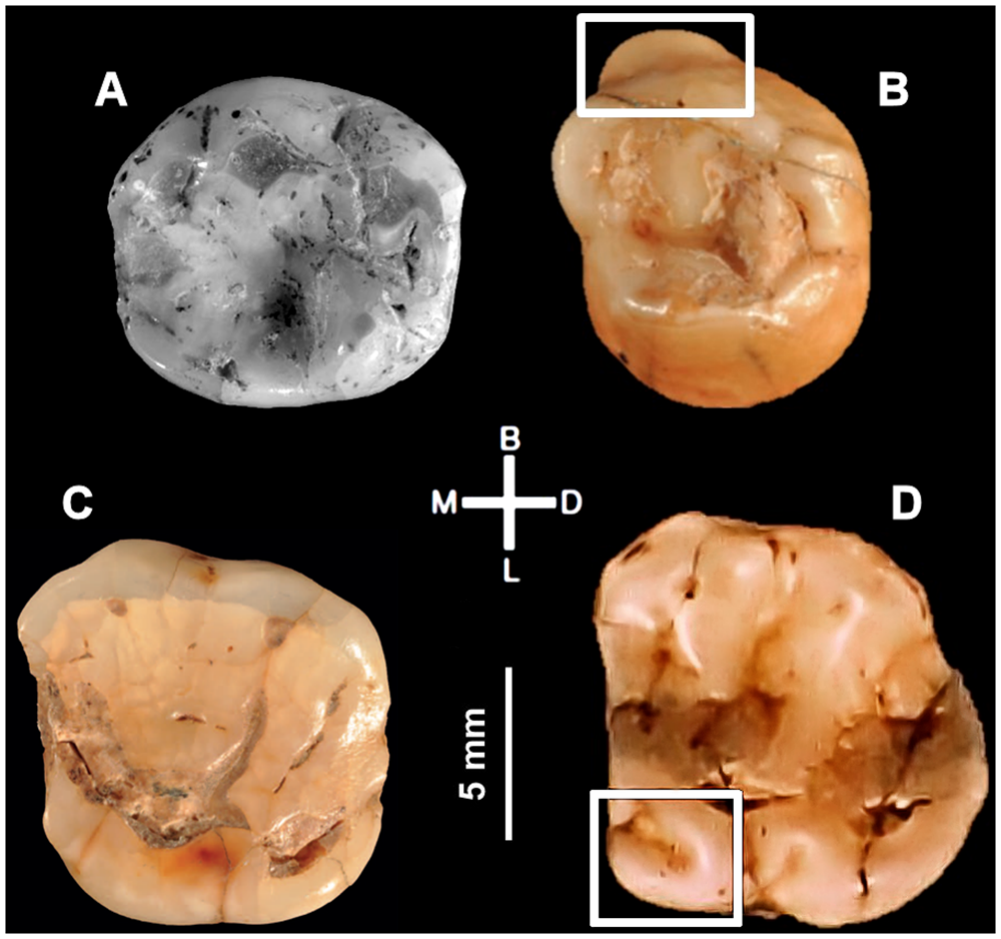
The Pleistocene human teeth from southern Italy discovered by A. Palma di Cesnola. **A.** left dM_2_ referred to layer L of Cavallo (the A-tooth); **B.** left dM^1^ referred to level E-III of Cavallo (the B-tooth); **C.** dM^2^ referred to level E-II/I of Cavallo (the C-tooth: left according to ref. [12], right according to ref. [15]); **D.** left M^1^ referred to layer 6 of Poggio (the D-tooth). The white rectangles mark the *tuberculum molare* of the B-tooth and the Carabelli cusp of the D-tooth. Provenience is according to refs. [2,12,54]. Photos by S. Benazzi and reproduced with permission.

The original study of the human teeth reported to come from the Cavallo Uluzzian [15] designated them as “B” (a left dM^1^) and “C” (a right dM^2^ that is elsewhere classified as left [12]). That study also analyzed another deciduous tooth reported to come from the underlying Mousterian—“A,” a left dM_2_ from layer L. A fourth tooth described alongside, “D,” is an adult left M^1^ reported to come from the Mousterian levels of Grotta del Poggio, at Marina di Camerota (Salerno), a.k.a. Grotta “de li Poggi” [15,52]. These four teeth were again published a decade later, with no significant modification of the analysis [53]. Here, they will be referred to using the original designations, expressed as follows: A-tooth, B-tooth (“Cavallo-B” in ref. [12]), C-tooth (“Cavallo-C” in ref. [12]), and D-tooth (Fig 11).

Where the A-tooth is concerned, the stratigraphic provenience above is corroborated by the information given in the 1965 field report: “Of great interest is the recovery of a human molar in the lowermost spit of layer L” [22] (p. 292). The B-tooth and the C-tooth are mentioned in the more recent literature [2,12] as coming, respectively, from E-III and E-II/I. Their primary anthropological description provides no year-of-excavation information and only states the following: “We ought to specify that the B-tooth was retrieved in the first Uluzzian *foyer*, which directly overlies the last Mousterian soil ‘F.’ The C-tooth, although belonging to the same cultural horizon, comes from a point situated some 15–20 cm above” [15] (p. 251). A decade later, the same authors [53] assign the C-tooth to the E5 spit but do not specify whether this assignment placed it in E-III or in E-II/I. From the thickness of E-III as represented in the published schematic profiles (Fig 3), and the “some 15–20 cm above” elevation reference, either is possible; however, the E5 spit is explicitly assigned to level E-III in Palma di Cesnola’s monographic study of the stone tool assemblages [23] (p. 54).

Subsequent writings are also consistent with the notion that the C-tooth came from a level E-III, not a level E-II/I spit: “At Grotta del Cavallo, during the 1964 excavations, two human teeth were recovered in the deepest part of the Uluzzian deposit, more precisely in **level E7 (base of the Archaic Uluzzian horizon**), and in **a level of the same cultural horizon** situated 15–20 cm above” [3] (p. 806, in French) [4] (p. 115, in Italian) (our emphasis). Palma di Cesnola consistently refers to E-III as the “Archaic Uluzzian” **horizon** in which both teeth would have been found, while, to him, level E-II/I represented a “Middle Uluzzian” **horizon**. With respect to the latter, no mention exists in the 1960s annual field reports of human teeth having been found therein.

This information suggests that subsequent commentators may have been misled into thinking that the C-tooth belonged in E-II/I by the “some 15–20 cm above” elevation description, and serves to place the C-tooth alongside the B-tooth, both in E-III. Indeed, among the remarkable finds made in 1964, the corresponding field report mentions the discovery in level E-III of “some” human teeth [20] (p. 27). However, we know that among the 1964 finds from E-III there should also be a deciduous “Neandertal” incisor recently reported to be non-human [16,17,43,55]. The finds that Palma di Cesnola refers to in 1964 could therefore consist of (a) the B-tooth and that incisor, (b) both plus the C-tooth, (c) the C-tooth and the incisor, or (d) given the meaning of *alcuni* (“a small but indefinite amount”), the Italian word actually used, any of these plus additional material subsequently lost or recognized as non-human.

Another complicating factor is the statement concerning level D-I made in the report on the first Cavallo field season (1963): “this horizon also yielded a human milk tooth” [19] (p. 54). Bearing in mind the indistinct interface between layers D and E and the absence of human fossils in the original field reports’ descriptions of the contents of level E-II/I, it remains possible, therefore, that (a) the C-tooth found stratigraphically above the B-tooth is in fact the layer D fossil, (b) the excavator’s 1989 reference to 1964 instead of 1963 as the year of discovery represents a lapse of memory, and (c) he either changed his mind on the fossil’s stratigraphic context or mixed it up by accident (and we do know that items whose ink-marking indicates provenience from layers B and D were subsequently assigned to layer E; see above).

More troubling is the unambiguous reference to morphology that, in the 1964 report, accompanies the mention of human tooth finds: “among which, an infantile molar (now under study at the Institute of Anthropology in Florence) presenting a pronounced Carabelli cusp” [20] (p. 27). However, no such tooth exists among the four studied by Palma di Cesnola and Messeri [15,53]. The A-, B- and C- teeth are deciduous molars but only B- and C- are maxillary, making them the only ones on which a Carabelli cusp could be present but neither bears one (Fig 11). In the case of the C-tooth, if it initially possessed a slightly expressed Carabelli feature, then evidence of its presence would have been erased by the heavy wear of the crown, so none could have been diagnosed in the first place; conversely, had a developed Carabelli existed, the tooth’s advanced wear should make it apparent in occlusal view, but this view shows no such feature. The D-tooth is also a maxillary molar, and does have a clear Carabelli cusp, but it is an adult permanent tooth reported to come from a different site, the Grotta del Poggio.

Clearly, there is an error here, but where? There are two possibilities: when describing the E-III tooth from 1964, the excavator (a) misidentified the type of feature present on that dental remain as a Carabelli cusp instead of the *tuberculum molare* found on the B-tooth, or (b) identified a Carabelli cusp on a tooth recovered in 1964 that at the time he saw as human but is neither the B-tooth nor the C-tooth. Determining which is the more likely would be difficult and does not directly bear on our focus on site stratigraphy and assemblage integrity. The key point here is that, given the uncertainty, the text of the original field report is insufficient to corroborate that the B-tooth and the C-tooth of the 1967 description are those (or among those) found in the 1964 excavation of level E-III.

In summary, the primary sources disagree as to the exact stratigraphic position of the B-tooth and the C-tooth within the Uluzzian of Cavallo. The first has been assigned to E-III and the second to E-II/I, but the primary description of the remains in 1967 points to both being from E-III. This, however, leaves the human milk tooth from D-I mentioned in the 1963 field report unaccounted for. In addition, no tooth exists among those reported in the 1967 study that matches the description of the one Cavallo tooth for which informative detail is provided in the original field reports—the “infantile molar presenting a pronounced Carabelli cusp” from 1964. Qualifying as uncertain the provenience of the human remains heretofore associated with the Uluzzian from Cavallo is justified and, in the very least, an understatement.

### Summary and implications

We have established that the artefact assemblage assigned to layer D of Cavallo is primarily composed of Protoaurignacian and Aurignacian lithics. We have also shown that objects of earlier (Uluzzian) and later (Early Epigravettian) age are present in the layer D assemblage as a consequence of excavation error and/or post-depositional disturbance. The fact that post-depositional disturbance caused extensive damage to the Upper Paleolithic deposit of Cavallo was eventually recognized (and acknowledged) by Palma di Cesnola; regrettably, this only happened at a time when most of the layers that he assigned to the Uluzzian (layer D and layer E) had already been excavated.

There are clear indications that, in the wake of such recognition, Palma di Cesnola carried out a measure of post-hoc “correction” of the field provenience of certain items. We have documented re-assignment to layer E of material ink-marked with layer B and layer D codes, as well as the inclusion in the layer D assemblage of finds that, in fact, came from disturbed deposits.

The provenience of the radiocarbon-dated ornamental shell samples is also equivocal. If they all come from the 1963–64 trench, then the disturbance affecting significant portions of layers D and E of that trench and the instances of post-hoc stratigraphic re-assignment of artefacts mean that the individual results obtained for the dated samples can only inform us about the respective age of each; those results would corroborate the presence of Early Epigravettian, Aurignacian, Protoaurignacian, Uluzzian and Mousterian material in layers D and E but, since the contextual uncertainty precludes dating by association, they would be mute as to the real age of any other items truly or putatively found alongside. If, as claimed [12], the layer E samples all come from in situ deposits excavated in 1978–84, then they would serve to constrain the age of the Uluzzian and the Aurignacian at the site; but they would remain of no relevance for the association-dating of the human fossils because, in that case, they would have come from different parts of the cave, ones whose stratigraphic configuration was distinct from that pertaining where (the 1963–64 trench) the B-tooth and the C-tooth were found.

If layer D is the correct provenience of one of the “Uluzzian” teeth, as we would be led to believe on the basis of the 1963 excavation report, then the default cultural attribution of such a tooth is the Protoaurignacian or the Aurignacian, even though the Gravettian or the Early Epigravettian are also distinct possibilities; in fact, the only two radiocarbon samples provenanced to the 1963–64 trench beyond reasonable doubt— (Cvl 10, OxA-21072, 19,685 ± 75 BP; Cvl 11, OxA-20631, 36,780 ± 310 BP)—were dated to these periods, not to the Uluzzian. If we admit a level E-II/I provenience for the C-tooth, as claimed in later reports, then the presence in that level of Dufour bladelets, coupled with the indistinctive nature of the boundary with overlying layer D, means that this tooth’s appurtenance to the Protoaurignacian or the Aurignacian cannot be excluded.

If the B-tooth and the C-tooth do come from the 1964 excavation of level E-III, our reconstruction of the site’s excavation history implies a strong presumption that they could have come from a disturbed area. This is because it was not until their would-be year of discovery that the excavator became aware of the pit cutting the excavation trench to the top of layer F, and not until much later that the real, much larger extension of that pit could be recognized. Therefore, like those from 1963, the 1964 finds must include items retrieved in areas affected by this major disturbance feature.

To counter this conclusion, one could argue that, on the basis of published records, the contamination with non-Uluzzian lithics of the lower part of layer E of the 1963–64 trench is, by comparison, minor, and, therefore, that the B-tooth and the C-tooth, if from E-III, are unlikely to be intrusive. However, bearing in mind (a) the presence of *Dentalium*, *Columbella* and *Cyclonassa* shells in layer F of 1963, (b) the arbitrary nature of the separation between E-III and E-II/I, and (c) the “corrected,” post-hoc nature of the stratigraphic assignment of at least some of the material in the assemblages from layers D and E, it is clear that little confidence can be placed in such a line of counter-argumentation. Furthermore, even though intrusions to a comparable depth were not apparent among the deposit’s larger-sized items, smaller-sized ones, such as human milk teeth, perfectly well could have been post-depositionally taken down from layer B or layer D to the same depth as the shell ornaments reported from layer F—as indeed corroborated by the presence of microlithic Dufour bladelets in level E-III.

Knowing how cave sites were excavated at this time, it is hardly surprising that the Cavallo assemblages suffer from major problems of stratigraphic integrity. From the coeval literature, it is apparent that the standard approach consisted of: firstly, opening a narrow, deep stratigraphic recognition trench; secondly, extending the excavation to adjacent blocks, sideways from that trench, and advancing through horizontal spits of *a priori*-defined, arbitrary thickness, with no piece-plotting of individual finds; thirdly, linking the different spits, on the basis of depth information, to each of the units recognized in the trench walls extant at the end of each season, when stratigraphic cross-sections were again described. It is easy to see how such a *modus operandi* would not have been very effective in detecting disturbance features during the course of excavation, especially ones as large as the Cavallo pit; this is something that can only be adequately done in the framework of the *décapage* (i.e., synchronic area excavation) of extensive paleosurfaces. Indeed, once the excavator exposed an area sufficiently large for the pit at the center of the cave to be recognized, the disturbance was identified; alas, that was too late to exempt from significant uncertainty the original stratigraphic position of the finds of key paleoanthropological importance.

Major post-depositional disturbance also afflicted the other Uluzzo Bay sites—Cavallo is in no way exceptional in this regard. Palma di Cesnola speculated that the pit resulted from deliberate excavation by the site’s Romanellian occupants [24], and, more recently, its origin has been attributed to the operation of subsidence processes [33]. We believe, however, that it more likely relates to the past activities, over decades or centuries, of local treasure hunters—as suggested for Grotta di Uluzzo and Grotta-Riparo di Uluzzo C by their excavator, Borzatti von Löwenstern [56–58]. He reports such activities to have almost completely destroyed the upper part of the Uluzzo and Uluzzo C sequences down to at least some 2 m below the surface. We find it hard to believe that something similar did not occur at the neighboring Grotta del Cavallo, which is about the same size and as easy to access as the other two (Fig 2).

For discussions concerning the authorship of the technocomplex, the taxonomic affiliation, modern human or Neandertal, of the teeth from the Uluzzian of Cavallo, is, in this context, a moot point. Where the value of these remains primarily lies is in reminding us yet again that extreme caution is warranted when assessing the significance of small, isolated, and undated human fossils. This point had already emerged from numerous examples of late Middle or early Upper Paleolithic contexts once thought to be as straightforward as portrayed in recent accounts of the Cavallo stratigraphic sequence; when directly dated, many such “early modern humans” turned out to be historic, Neolithic or later Upper Paleolithic in age [59], while “Neandertal” remains such as the San Bernardino specimen turned out to be medieval modern human [60].

Despite this significant and growing corpus of cautionary tales, a salient feature of some recent accounts of the Middle-to-Upper Paleolithic and Neandertal-to-modern human transitions in Europe [12,49,61,62] has been their reliance on a very limited number of fragmentary fossils from problematic contexts: namely, the Kent’s Cavern maxilla and the two Cavallo teeth. The weight of the data since brought to bear on the formation process and excavation history of Kent’s Cavern [16,63] has made it clear that such evidence is also characterized by a dubious stratigraphic context, and this is now equally clear in the case of Cavallo. Indeed, in their most recent synthesis [64], supporters of the relevance of Kent’s Cavern with respect to the timing of modern human entry into Europe no longer take the fossil into consideration; it is only on the evidence from Cavallo that they hang the notion that the symbolic aspects of late Neandertal culture either result from “acculturation” through contact with coeval, nearby modern human populations [8,64] or reflect the mistaken attribution of the Châtelperronian, Uluzzian and other transitional complexes to Neandertals instead of to modern humans [11–13,17,62,64]. However, the research we report here shows that, at Cavallo, the context of the relevant fossils, even if better than at Kent’s Cavern, cannot be used to make inferences concerning their age and cultural affiliation.

The uniting motto of such recent dating research [64] has been to place modern humans in Europe earlier than previously thought, and to infer from such a revised chronology that (a) modern humans were the authors of the transitional technocomplexes, and (b) the archaeology Neandertals are associated with is pre-symbolic. With this in mind, it is important to note that, at Cavallo, the issues of assemblage integrity affecting the human remains extend only in part to the evidence concerning the symbolic aspects of Uluzzian material culture because two of the directly dated ornamental marine shells yielded ages in excess of ca. 39–40 ka ^14^C BP (ca. 43–44 ka cal BP). These *Dentalium* tubes fall within the time range of the Uluzzian elsewhere in Italy even if the reservoir effect and a potential lag of some two millennia between age of the shell and time of human collection and use are accounted for (Fig 9). They therefore can be added to the growing body of evidence concerning symbolism in the pre-Aurignacian archaeological record of Europe [7,9,65–72].

The Kent’s Cavern and Cavallo cases highlight how the radiocarbon dating of samples obtained from old museum collections lacking adequate information pertaining to their stratigraphic and taphonomic context is unlikely to solve complicated association problems and may simply serve to further muddy the waters. When the integrity of the assemblages is warranted and the relevant field information exists, many examples show that their critique with analytical approaches such as lithic taphonomy can move the field forward. This is how the long-standing controversy surrounding the putative interstratification of Aurignacian and Châtelperronian occupations at Le Piage, Roc-de-Combe and Grotte des Fées eventually was resolved [73,74]. In the Cavallo case, consideration of the spatial distribution of finds across the sectors into which the excavator divided the 1960s trench might have enabled sorting into sub-assemblages defined according to the likelihood of their coming from areas affected by the large pit disturbance first identified in 1964. This would have been especially useful for the human teeth, due to the role they have come to play in debates concerning the spread of modern humans into Europe. However, such spatial information has never been published and, if at all noted at the time of digging, the corresponding records are wanting. Furthermore, the Cavallo collections have since undergone considerable losses, namely that of most of the debitage [36] (p. 75).

Direct dating of the Cavallo remains might dispel doubts but has been made impossible by the removal of the fossils’ dentine [75]. Since, as reviewed here, little confidence can be placed in the provenience and stratigraphic context reported for them, it follows that the Cavallo teeth cannot be used in any reliable manner to argue one way or the other about the authorship of the Uluzzian and ought to be left out of discussions concerning the makers of the archeological cultures involved in the Neandertal-to-modern human transition.

## Conclusion

The contextual weaknesses of the Cavallo evidence mean that the authorship of the Uluzzian can only be addressed indirectly, via due consideration of wider spatiotemporal distribution patterns—under an allopatric understanding of Neandertals and modern humans, the former must be the makers of Europe’s archeological record prior to the time when presence of the latter is first recorded in the continent. After more than 150 years of intensive research, that time is no earlier than 41.4 ka cal BP, the lower limit of the 95.4% probability interval of the direct radiocarbon date obtained on the Oase 1 mandible (OxA-11711/GrA-6165: 34,950/+ 990/-890 BP) [76]. In terms of first settlement dating, this result might be considered as just a minimum age. However, under the allopatry assumption and the consensus view that the spread of modern humans into Europe unfolded from East to West, modern humans cannot have been in Italy at a time when direct dating of diagnostic remains shows that adjacent lands situated across the Alps and the Adriatic Sea remained Neandertal territory; and this is indeed the situation observed ca. 45 ka cal BP, when the Uluzzian began [12,17].

**Fig 12:**
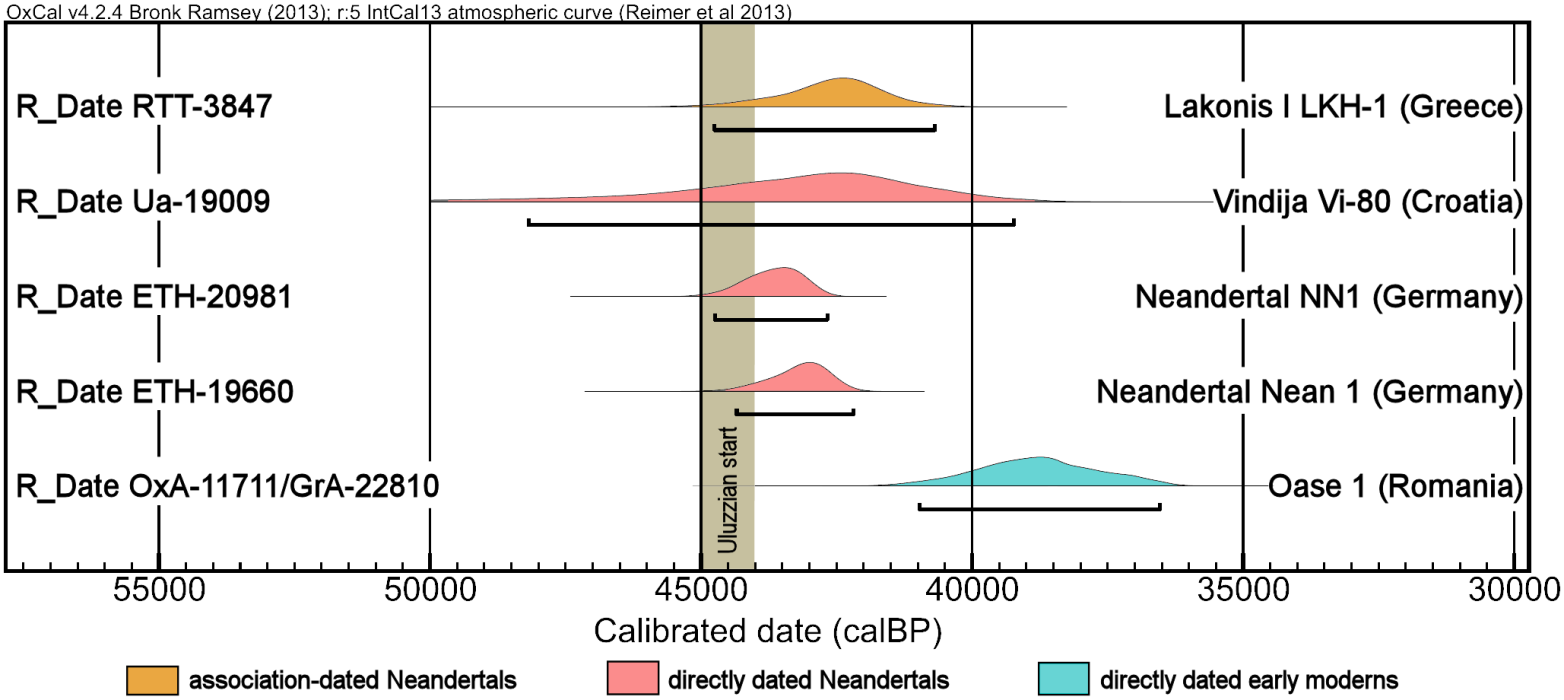
Neandertals from the time of emergence of the Uluzzian and age of Europe’s earliest moderns. The distributions correspond to the 95.4% probability intervals of radiocarbon dates calibrated with the INTCAL13 curve using OxCal v.4.2.4 [81,82]. The start date for the Uluzzian is that obtained for the site of Fumane, the best constrained of those modelled in ref. [17]. The LKH-1 Neandertal was found 20 cm above the charcoal sample that yielded the RTT-3847 result, which therefore represents the fossil’s maximum age [77]. The dates for the Oase, Vindija and Neandertal fossils are drawn from refs. [76,78,79]. When the Uluzzian emerged in Italy, the adjacent land to the East, Northeast and North was inhabited by Neandertals, not modern humans.

As illustrated in Fig 12, the emergence of the Uluzzian (a) to the East, overlaps the time range obtained for the accumulation of the deposit containing the Neandertal molar from Lakonis I, Greece [77], (b) to the Northeast, dates to the lower limit of the 95.4% probability interval obtained for the age of the Vi-80 Neandertal fossil from Vindija, in Croatia [78], and (c) to the North, predates the lower limit of the 95.4% probability intervals obtained for the Nean 1 and NN1 fossils from the eponymous site in Germany [79]. In addition, no crossings of the Central Mediterranean by sea are documented prior to ca. 7,000 years ago, and the islands of Sicily, Corsica or Sardinia remained uninhabited until the end of the Upper Paleolithic or the Mesolithic. Indeed, no connection between the Uluzzian and the material culture of coeval early modern humans in Egypt or the Maghreb exists or has ever been proposed to exist. Conversely, use of the Levallois reduction method—altogether abandoned in the Aurignacian and all other technocomplexes of the Early Upper Paleolithic made by modern humans—establishes an unambiguous technological and cultural link between the Uluzzian and the preceding, Neandertal-associated Mousterian. That Neandertals made the Uluzzian [80] therefore remains the parsimonious reading of the evidence.

## Materials and Methods

The key source of historical information concerning Italy’s Paleolithic Archeology of the 1960s and 1970s is the University of Florence *Rivista di Scienze Preistoriche*, which is available in libraries worldwide. When contacted, Apulia’s Direction of Antiquities replied that no unpublished excavation reports, diaries or field notes concerning the work carried out at Grotta del Cavallo by Arturo Palma di Cesnola in the 1960s and 1970s exist in its archive.

The unavailability of excavation records and the lack of suitable stratigraphic and topographic information (e.g., site plan, site grid, detailed stratigraphic descriptions, or spatial distribution data) precluded use of standard archeological techniques for the assessment of issues pertaining to site formation and taphonomy. We addressed these problems using a historiographic/philological approach based on the examination and critical reading of coeval sources. The potential of this strategy to advance important debates in paleoanthropological research is highlighted by the cases of the Grotte des Fées at Châtelperron [83] and Kent’s Cavern [63].

In addition, three of us (JZ, WB, FD) directly examined all the archaeological material from Palma di Cesnola’s 1960s Cavallo excavations that, as of June, 2014, was in storage at the Taranto depot of Apulia’s Direction of Antiquities (*Magazzini Materiale Archeologico*, Complesso demaniale di S. Antonio, Via Viola 12, Taranto, Italy). An inventory (the *schede*) elaborated in the early 1970s by archeologist Mariantonia Gorgoglione and containing detailed descriptions of the material from the 1963–64 excavations that was transferred to Taranto exists in the head office of the agency (*Soprintendenza per i Beni Archeologici della Puglia*, Via Duomo 33, 74100 Taranto, Italy), where it can be consulted.

We compiled a database with the contextual, techno-typological and photographic information derived from our examination of the Taranto material and inventory. In order to further our analysis of assemblage integrity, this database was then compared with (a) the contents of the complete corpus of publications referring to or reporting on the site and its finds, as well as (b) the contents (artefact records noting ink-marked code and including the corresponding description and drawing) of the inventory produced by the other of us (PG) in 1985–86 while researching the Cavallo lithics for a Ph.D. dissertation on the Uluzzian [35] (S3 Appendix).

## Supporting Information

S1 Appendix Contents of bag No. 396 of the Grotta del Cavallo collection, paper-labeled “F I/II,” in storage at the Taranto depot of Apulia’s Direction of Antiquities. Flakes, sidescrapers and fragments thereof, all made on siliceous limestone and/or *lastrine*, and all labeled “1U57A.”
(TIF)

S2 Appendix *Cyclonassa* shells from the Grotta del Cavallo collection in storage at the Taranto depot of Apulia’s Direction of Antiquities. Note the identical placement and type of perforation apparent in all the shells, irrespective of assigned stratigraphic provenience. Note also the apparently identical texture and color of the sedimentary matrix observed in specimens assigned to levels as high up in the sequence as B-IIa and as low down in it as D-II, in agreement with the level descriptions and the University of Florence’s particle-size analysis of sediments, published with the 1963 excavation report [15] (p. 44–45).
(TIF)

S3 Appendix Patrizia Gioia’s 1985–86 inventory of the stone tools from layers D and E of Grotta del Cavallo. Reproduction of one of the recording sheets produced, illustrating the nature and quality of the information (number of drawn item, Bordesian type-list number, ink-marked code, description, drawing, blank type, dimensions, raw-material and striking platform).
(TIF)
