# Supplementary figures and images for: Analysis of Site Formation and Assemblage Integrity Does Not Support Attribution of the Uluzzian to Modern Humans at Grotta del Cavallo
Source: PLoS One. 2015 Jul 8;10(7):e0131181. doi: 10.1371/journal.pone.0131181 (PMC4495988; doi:10.1371/journal.pone.0131181)

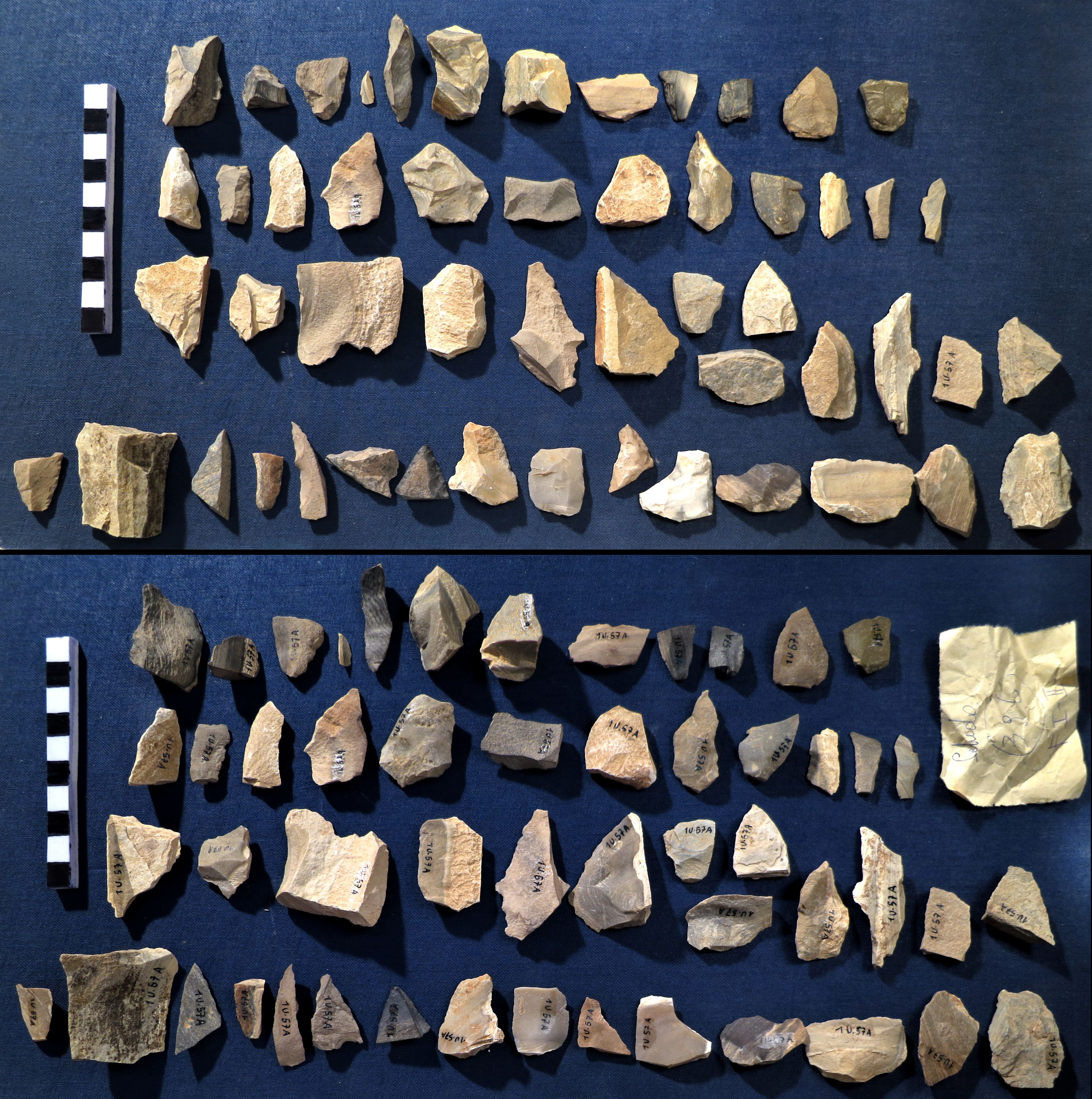

Supplement: S1 Appendix — Flakes, sidescrapers and fragments thereof, all made on siliceous limestone and/or lastrine, and all labeled “1U57A.” (TIF) [file pone.0131181.s001.tif]

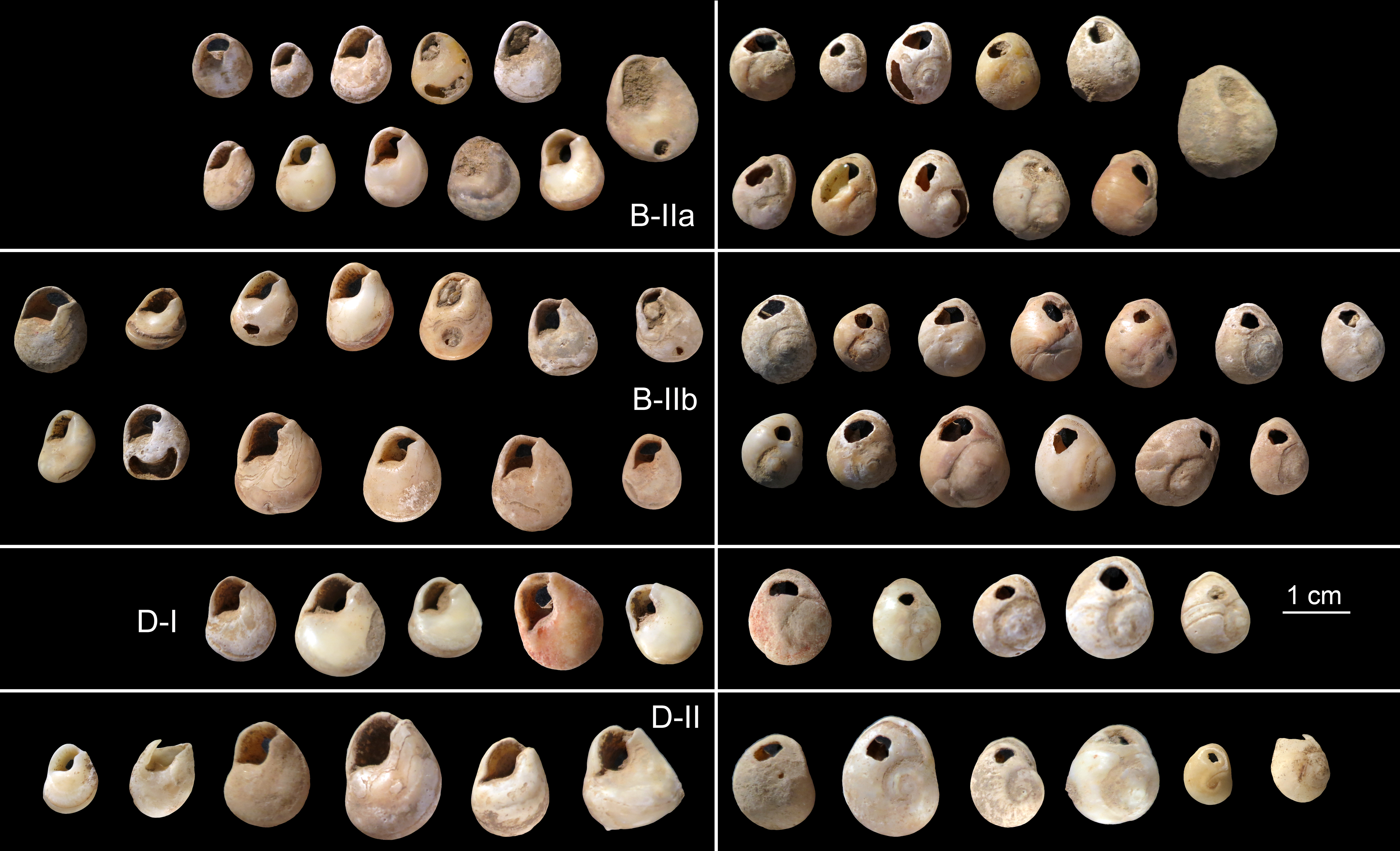

Supplement: S2 Appendix — Note the identical placement and type of perforation apparent in all the shells, irrespective of assigned stratigraphic provenience. Note also the apparently identical texture and color of the sedimentary matrix observed in specimens assigned to levels as high up in the sequence as B-IIa and as low down in it as D-II, in agreement with the level descriptions and the University of Florence’s particle-size analysis of sediments, published with the 1963 excavation report [15] (p. 44–45). (TIF) [file pone.0131181.s002.tif]

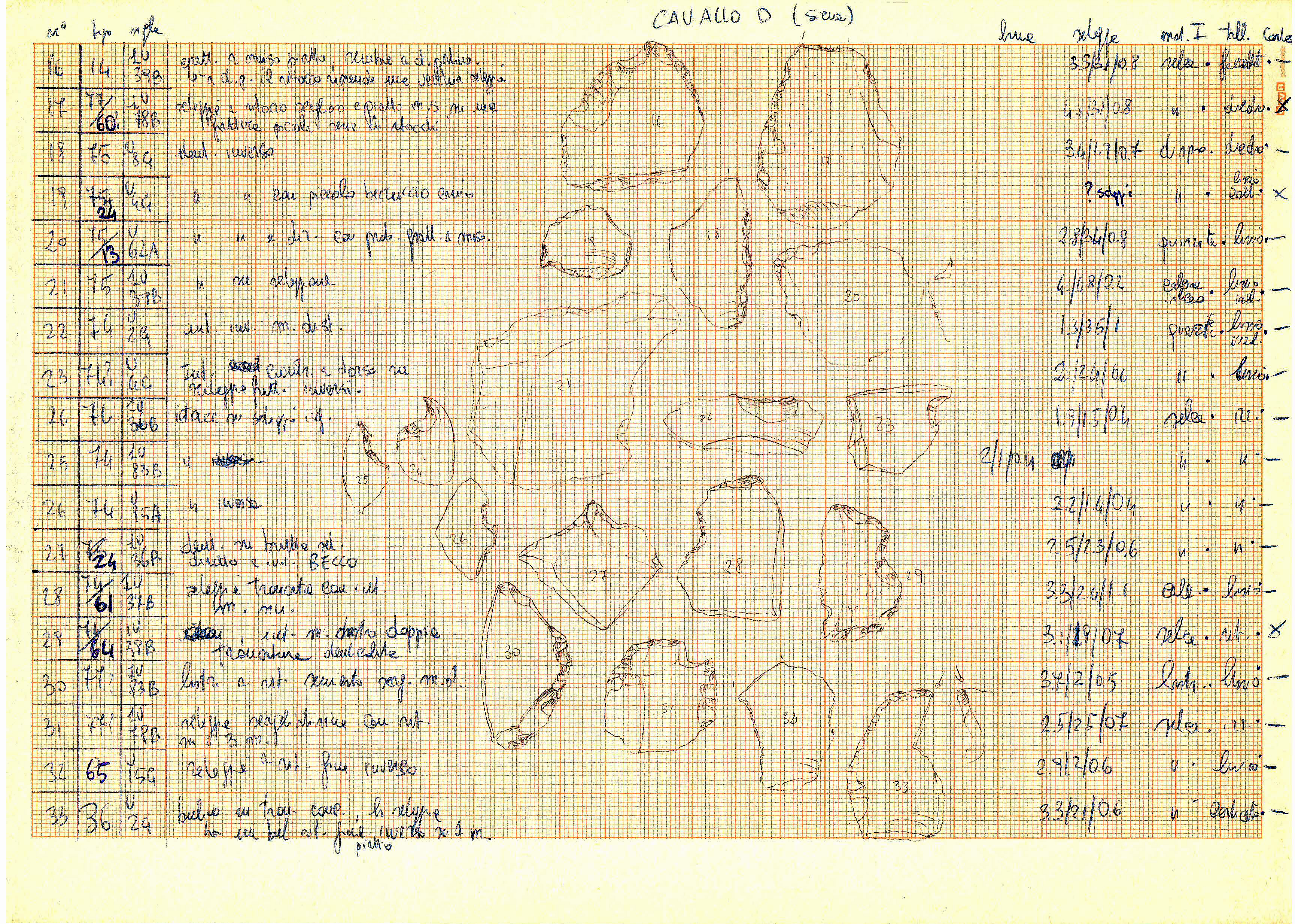

Supplement: S3 Appendix — Reproduction of one of the recording sheets produced, illustrating the nature and quality of the information (number of drawn item, Bordesian type-list number, ink-marked code, description, drawing, blank type, dimensions, raw-material and striking platform). (TIF) [file pone.0131181.s003.tif]
